# Comparison of multiple antigens for use in indirect ELISAs for detection of anti-capripoxvirus antibodies in sheep, goats, and cattle

**DOI:** 10.1128/jcm.01191-25

**Published:** 2026-06-12

**Authors:** Mahder Teffera, Leanne McIntyre, Shawn Babiuk

**Affiliations:** 1 Canadian Food Inspection Agency, National Centre for Foreign Animal Disease 558430, Winnipeg, Manitoba, Canada; 2 Department of Immunology, Max Rady College of Medicine, University of Manitoba 8664, Winnipeg, Manitoba, Canada; University of California Davis, Davis, California, USA

**Keywords:** capripoxvirus, goat pox, lumpy skin disease, sheep pox, ELISA

## Abstract

The virus neutralization test (VNT) is currently the most appropriate diagnostic test to detect antibodies specific to sheep pox, goat pox, and lumpy skin disease virus. There is one commercial enzyme-linked immunosorbent assay (ELISA) available; however, this ELISA has some limitations. Several antigens have been identified as possible targets but have not been compared and assessed with the virus neutralization test and commercial ELISA. Improving serological testing for capripoxviruses is required to improve control of capripoxviruses and to demonstrate freedom from infection.

## INTRODUCTION

The genus *Capripoxvirus* of the Poxviridae family consists of sheep pox virus (SPPV), goat pox virus (GTPV), and lumpy skin disease virus (LSDV). There is a high genetic similarity between the viruses, displaying over 97% sequence identity (1). Capripoxviruses exhibit host specificity, with SPPV primarily infecting sheep, GTPV primarily infecting goats, and LSDV infecting cattle (2). However, some SPPV and GTPV isolates can cross-infect both sheep and goats (3).

Together, capripoxviruses cause economic losses to the sheep, goat, and cattle industries where these viruses are present and continue to pose a global threat (4, 5). SPPV and GTPV are endemic in most of Asia, the Middle East, and Africa, excluding southern Africa. There have also been outbreaks in Spain, as well as Greece and Bulgaria (6). The latest SPPV/GTPV outbreaks were reported by Romania and Serbia, in June and September 2025, respectively (7). LSDV is endemic throughout Africa, with the latest outbreaks reported in Algeria, Tunisia, and Libya, leaving Morocco as the last LSDV-free country in Africa. LSDV is also endemic in the Middle East and throughout Asia, with recent expansion into Indonesia and outbreaks in Korea and Japan (8). In July 2025, lumpy skin disease (LSD) caused outbreaks for the first time in Italy and France, possibly due to spread from North Africa via vectors (9). This has since extended to Spain, with more cases reported in France (10). SPPV and GTPV are associated with high rates of mortality and morbidity, reported as high as 90% and 100%, respectively (2, 4). In contrast, LSDV is less severe, with a variable rate of morbidity of up to 50% and a mortality rate ranging between 1% and 10% (4). Skin lesions accompanied by fever are the characteristic clinical signs of the disease. Diagnosis of capripoxviruses requires laboratory confirmation, which can be performed using various diagnostic tests, including molecular tests (e.g., PCR), serological tests, electron microscopy, or virus isolation (2).

Serology can be performed using the virus neutralization test (VNT), immunoperoxidase monolayer assay (IPMA), or enzyme-linked immunosorbent assay (ELISA) (11). Although the VNT is considered a sensitive test and a gold standard, it suffers from being complicated, labor-intensive, and time-consuming. Furthermore, it requires a high containment laboratory to perform as live virus is utilized—a limitation also shared with performing IPMA. There is a commercial ELISA available; however, this test is not fully validated, and its diagnostic sensitivity and specificity remain to be comprehensively assessed. Reports of varying success using the commercial assay highlight a need for the development of a quick, robust, and consistent serological assay for capripoxviruses (12–14).

Several different ELISAs have been developed to detect anti-capripoxvirus antibodies using either purified inactivated virus (15) or recombinant proteins, including 95 and 103 (16), 74 (p32) (17–19), 60 (L1R) (20), 117 (A27L) (21, 22), 122 (A32R) (23–26), 123 (A34) (12), and B22R (27). Inactivated virus ELISAs are problematic due to the time and labor cost of the production of the antigen, which requires growing large amounts of virus (28). Determining which antigen or antigens are the most effective for use in an ELISA is a critical gap in the development of validated ELISAs for SPPV, GTPV, and LSDV (4, 19, 29). The antigens used in this study were selected based on the immunogenicity of vaccinia virus homologs, antigenicity prediction, and previous studies describing the antigens in capripoxviruses (29).

Soluble baculovirus-expressed proteins: LSDV 60 (L1R), LSDV 74 (p32), LSDV 117 (A27L), LSDV 122 (A33R), LSDV 123 (A34), and LSDV 141 (B5R) were selected as potentially immunogenic surface proteins and evaluated as antigens in indirect ELISAs (iELISA) to detect anti-capripoxvirus antibodies in experimentally infected sheep, goats, and cattle.

## MATERIALS AND METHODS

### Expression construct design

Consensus sequences of six open reading frames (ORF) were generated from nucleotide sequences of each gene using SPPV, GTPV, and LSDV genomes from GenBank (National Center for Biotechnology Information) based on a majority-rule approach at each nucleotide position, given the high degree of nucleotide and amino-acid conservation in capripoxviruses (1, 30). The ORFs are LSDV 60 (L1R), LSDV 74 (p32), LSDV 117 (A27L), LSDV 122 (A33R), LSDV 123 (A34), and LSDV 141 (B5R), ordered based on their position on the LSDV genome. The accession numbers of the sequences used to make a consensus are NC_004002 (SPPV), MW020571 (SPPV), MT137384 (SPPV), MN072626 (SPPV), MW020570 (GTPV), MN072625 (GTPV), MN072620 (GTPV), KC951854 (GTPV), MZ577073 (LSDV), MW355944 (LSDV), MT134042 (LSDV), MK441838 (LSDV), and AF325528 (LSDV). The consensus sequence was then translated using Geneious Prime 2021.0.3 to predict the topology using CCTOP v1.1.0 and deepTMHMM v1.0.19. The transmembrane and internal regions were removed from the nucleotide sequence. Sequences were modified to remove the start and stop codon if present, and restriction sites were included (NotI and BamHI) to allow for cloning into an expression plasmid: pAB-Bee-FH (AB Vector, catalog number #B3FH). While the removal of transmembrane regions improves solubility, it may result in alteration of the native conformation of epitopes with a potential impact on antigenicity (31, 32). LSDV 117 (A27L) was not predicted to contain a transmembrane region using CCTOP v1.1.0 and deepTMHMM v1.0.19; therefore, the full sequence was expressed. The nucleotide sequences and plasmids were provided to a commercial supplier (Biomatik) to synthesize the sequences and clone them into the plasmid.

### Protein production

Expression plasmids and baculovirus (ProFold-ER1, AB vector, catalog number #A4) for each protein were co-transfected into *Spodoptera frugiperda* (SF9) insect cells according to the manufacturer’s protocol. Following co-transfection, baculovirus expressing each recombinant protein was propagated in SF9 cells to increase the virus titer. Protein expression was confirmed through the expression of a marker protein (GFP, green fluorescent protein) and Western blot analysis using anti-Histidine (His) antibody. Baculovirus expressing proteins of interest was then used to infect *Trichoplusia ni* (Tni) cells at a multiplicity of infection >5. Infected Tni cells were harvested after 2–4 days based on the presence of greater than 90% fluorescent cells (expression of GFP) quantified using the Invitrogen Countess 3 FL automatic cell counter (ThermoFisher Scientific). Tni cell pellets were then collected by centrifugation at 2,000 rpm for 15 min at 4°C and stored at −80°C until protein purification (up to 1 month) and thawed only once immediately before purification.

### Western blot

The supernatant from the shaker cell culture (to confirm protein expression) or purified protein was mixed with 4× NuPAGE LDS sample buffer (ThermoFisher Scientific, catalog number #NP0007) and 10× NuPAGE sample reducing agent (ThermoFisher Scientific, catalog number #NP0004) and denatured at 72°C for 15 min, in accordance with the manufacturer’s recommendation for optimal denaturation and reduced hydrolysis in LSD sample buffer (33). Denatured samples were loaded onto Bis Tris Gels (ThermoFisher Scientific, catalog number #NP0322) using 1× NuPAGE MOPS SDS running buffer (ThermoFisher Scientific, catalog number #NP0001) and ran at 120–140 volts until adequate separation was observed (1–1.5 h). Gels were transferred onto PVDF membranes using an iBLOT2 dry blotting system (ThermoFisher Scientific, catalog number #IB21001) and then blocked for 1 h at room temperature with shaking (20 RPM) or overnight at 4°C in 5% skim milk in 0.01 M phosphate-buffered saline containing 0.1% Tween-20 (PBST). Blocked membranes were washed 3× with PBST for 5–10 min and then incubated for 1 h with HRP-Anti-His antibody (Roche Life Science Products, catalog #11922416001), followed by washing 3× with PBST for 5–10 min and detection using substrate (TrueBlue peroxidase substrate, Seracare, catalog number #5510-0030). Membrane images were acquired with the Gel Doc EZ Imager (Bio-Rad) using inverted contrast settings for clarity.

### Protein purification

Tni cell pellets were re-suspended in lysis buffer containing IPER Insect cell protein Extraction Reagent (ThermoFisher Scientific, catalog number #89802) with cOmplete EDTA-free Protease Inhibitor Cocktail tablets (Roche Life Science Products, catalog number #11873580001, according to the manufacturer’s protocol), 10 mM imidazole, and 0.16 M NaCl at a volume of 1mL lysis buffer per 1–2 × 10^7^ cells. Resuspended pellets were centrifuged at 15,000 × *g* for 15 min at 4°C, and then the supernatant was collected. All following protein purification steps were done on ice. The pH of the lysate was adjusted to 8 using Tris-HCl buffer. The lysate was then filtered using 0.4 μm followed by 0.2 μm polyether sulfone (PES) membrane filters.

#### Benchtop purification

Ni-NTA agarose (Qiagen, catalog number #30210) was prepared for binding according to the manufacturer’s protocol. The cell lysate was mixed with Ni-NTA agarose on a rotator at 4°C for 1.5 h and then centrifuged at 2,000 × *g* for 15 min at 4°C. Agarose was washed with His-wash buffer (0.05 M phosphate buffer, 0.5 M NaCl, 10% glycerol, and 0.02 M imidazole) by adding greater than 3× volume of buffer, vortexing to mix, and then centrifugation at 2,000 × *g* at 4°C for 3–5 min. The supernatant was removed, and agarose wash step was repeated until the protein concentration measured below the NanoDrop Spectrophotometer (ThermoFisher Scientific) detection limit (~0.09 mg/mL) (34) after two consecutive washes using NanoDrop Spectrophotometer (ThermoFisher Scientific, catalog number #ND-ONE-W). The protein was then eluted by adding a volume of elution buffer (0.5 M imidazole, 0.05 M phosphate buffer, 0.25M NaCl, and 10% glycerol) less than half of the initial Ni-NTA agarose used, mixing gently, and then centrifuging at 2,000 × *g* at 4°C for 3–5 min. The elution step was repeated until the protein concentration declined substantially to maintain protein concentration and reduce co-elution of impurities (~0.5 mg/mL) (35, 36). Eluted protein was stored at −80°C and quantified using a Pierce BCA protein assay kit (ThermoFisher Scientific, catalog number #A55864) according to the manufacturer’s protocol.

#### Fast protein liquid chromatography (FPLC)

HisTrap excel (Cytiva, catalog number #17371206) columns were used to purify protein on ÄKTA FPLC system according to the manufacturer’s protocol. Equilibration, wash, and elution buffer were prepared using the His buffer kit (Cytiva, catalog number #11003400) according to the manufacturer’s protocol. Proteins were eluted in one of three ways, without using any stabilizing agent (LSDV 122 [A33R]) or with 10% glycerol (all other antigens) to prevent precipitation. Eluted proteins were stored at −80°C and quantified using a Pierce BCA protein assay kit (ThermoFisher Scientific, catalog number #A55864).

### Serum samples

Negative sheep, goat, and cattle sera were collected from Canadian animals under routine surveillance programs. Sera from capripoxvirus experimentally infected cattle (37, 38), sheep, and goats (3, 39) were used for this study.

### Virus neutralization test

Ovine testis cell line (OA3.T) cells were seeded into 96-well plates to be confluent the following day (~80%–90% confluence). After confirmation of cell confluence, sterile 96-well plates were used to perform serial 2-fold dilutions of serum starting with a 1/10 dilution in Dulbecco’s modified Eagle medium (DMEM) (Cytiva, catalog number #SH30262.01) supplemented with 2% FBS and 1% penicillin/streptomycin. Serum was diluted in duplicate to a total volume of 100 μL per well. A control plate was also included for quality control. In the control plate, positive (serum from experimentally infected animals) and negative control sera were serially diluted to a total volume of 100 μL per well. A back-titration of virus was also included on the control plate to confirm TCID_50_. A dilution of capripoxvirus (Kenyan LSDV) to a final concentration of 1,000 TCID_50_/mL was prepared. One hundred microliters (100 TCID_50_/well) of virus was then added to all wells, except for cell control wells (six wells), which received 100 μL of media. The virus and serum mixture plate were then incubated for 1 h at 37°C with 5% CO_2_. Following incubation, the virus and serum mixture (total of 200 uL) was transferred to confluent OA3.T cell plates after removal of existing media. The cells were then incubated at 37°C with 5% CO_2_ for 7 days to observe cytopathic effect (CPE) using a light microscope. VNTs were assessed upon confirmation of the virus titer and lack of CPE in cell controls, as previously described (40).

### iELISA development and optimization

Checkerboard titrations for the ELISAs were performed using serial dilutions of the antigen, serum, and conjugate. This was performed with sheep serum, followed by goat serum and bovine serum to establish conditions (Table 1). Absorbance values were averaged, and then a sample to positive ratio percentage (S/P%) was calculated based on the absorbance of standard serum samples adjusted for background absorbance on each ELISA plate {(serum absorbance/positive control absorbance)*100}. Final bleeds from experimentally infected animals were used as positive controls, with pre-bleeds used as negative controls. Serum was heat-inactivated at 56°C for 30 min prior to dilution. Heat inactivation could potentially alter antibody activity; however, it is necessitated to eliminate complement activation and inactivate capripoxvirus in accordance with World Organisation for Animal Health (WOAH) recommendations (41, 42). The use of serum-based blocking buffers and commercial blocking agents was evaluated in ELISA optimization; however, skim milk was used as a blocking agent for all iELISAs, excluding bovine LSDV 117 (A27L) iELISA, which was optimized using casein blocking buffer. Negative samples tested to evaluate specificity were disease-free sero-surveillance sera from Canadian cattle, sheep, and goats. Positive samples tested to evaluate sensitivity were experimentally infected animal sera on or after 21 days post-infection. Following optimization of iELISA protocols, commercial blocking buffers were used to block according to the manufacturer’s instructions to assess the performance of the antigens under various blocking conditions based on improved discrimination between positive and negative serum controls. The buffers assessed were SuperBlock (ThermoFisher Scientific, catalog number #37515), StartingBlock (ThermoFisher Scientific, catalog number #37538), ELISA Assay Buffer (ThermoFisher Scientific, catalog number #DS98200), and Pierce Protein-Free Blocking Buffer (ThermoFisher Scientific, catalog number #37572) (Tables S1, S2 and S3).

**TABLE 1:** Optimized iELISA conditions with recombinant capripoxvirus antigens

		Antigen per well (ng)	Serum dilution factor	Conjugate dilution factor
Ovine iELISA	LSDV 122 (A33R)	83	25	25
	LSDV 60 (L1R)	390	25	25
	LSDV117 (A27L)	164	25	25
	LSDV 123 (A34R)	90	100	100
Caprine iELISA	LSDV 122 (A33R)	111	25	25
	LSDV 60 (L1R)	390	25	25
	LSDV117 (A27L)	164	30	30
	LSDV 123 (A34R)	180	100	100
	LSDV 74 (P32)	50	100	100
	LSDV 141 (B5R)	88	25	25
Bovine iELISA	LSDV 122 (A33R)	83	25	25
	LSDV 60 (L1R)	390	25	25
	LSDV117 (A27L)	82	25	25

### iELISA protocols

#### Ovine/caprine ELISA

Antigen diluted in 0.05 M carbonate buffer in a volume of 100 µL was added to each well of a 96-well plate and incubated overnight at 4°C. The 96-well plates were washed 5× with 0.01 M phosphate-buffered saline containing 0.1% Tween-20 (PBST). One hundred microliters of 5% skim milk powder (SMP) in PBST was then added to each well and incubated for 1 h at 37°C with shaking (400 RPM). After incubation, plates were washed 5× with PBST. One hundred microliters serum diluted in 2.5% SMP in PBST (dilution buffer) was then added to respective wells and incubated at 37°C for 1 h with shaking (400 RPM). Following incubation, plates were washed 5× with PBST. One hundred microliters of the diluted conjugate in 2.5% SMP in PBST (HRP conjugated Donkeyanti-Sheep IgG, ThermoFisher catalog number #A16041 for ovine iELISA and HRP conjugated Rabbit anti-Goat IgG, ThermoFisher catalog number #A18751 for caprine iELISA) was added to respective wells, and the plates were incubated at 37°C for 1 h with shaking (400 RPM). After incubation, plates were washed 5× with PBST. One hundred microliters of TMB substrate was then added to wells for detection (SureBlue TMB Microwell Peroxidase substrate, SeraCare catalog number #5120-0075). Plates were protected from light and incubated for 10 min at room temperature with shaking (400 RPM). After substrate development, 100 µL of TMB stop solution (SeraCare, catalog number #5150-0020) was added to corresponding wells. Plates were then agitated, and absorbance was read at 450 nm on a SpectraMAX Plus 384 plate reader using SoftMax Pro v7.0 software.

#### Bovine ELISA

Antigen diluted in 0.05M carbonate buffer in a volume of 100 µL was added to each well of a 96-well plate and incubated overnight at 4°C. The 96-well plates were washed 5× with PBST. One hundred microliters of 5% SMP in PBST for LSDV 122 (A33R) and 60 iELISAs or 1× casein blocking buffer (Millipore Sigma, catalog number #B6429) for LSDV 117 iELISA was then added to each well and incubated for 1 h at 37°C with shaking (400 RPM). After incubation, plates were washed 5× with PBST. One hundred microliters serum diluted in 2.5% SMP in PBST (LSDV 122 [A33R]; LSDV 60 [L1R]) or 1% casein blocking buffer (LSDV 117 [A27L]) was then added to respective wells and incubated at 37°C for 1 h with shaking. Following incubation, plates were washed 5× with PBST. One hundred microliters of the diluted conjugate in PBST (HRP-conjugated goat anti-bovine IgG, ThermoFisher catalog number #A18751) was added to respective wells, and the plates were incubated at 37°C for 1 h with shaking (400 RPM). After incubation, plates were washed 5× with PBST. One hundred microliters of TMB substrate was then added to wells (SureBlue TMB Microwell Peroxidase substrate, SeraCare catalog number #5120-0075). Plates were protected from light and incubated for 10 min at room temperature with shaking (400 RPM). After substrate development, 100 µL of TMB stop solution (SeraCare, catalog number #5150-0020) was added to corresponding wells. Plates were then agitated, and absorbance was read at 450 nm on a SpectraMAX Plus 384 plate reader using SoftMax Pro v7.0 software.

### ID screen capripox double antigen Multi-species

ID ELISAs were performed and analyzed according to the manufacturer’s protocol using all positive ovine, caprine, and bovine serum used in iELISA validation. Plate absorbance was read at 450 nm on a SpectraMAX Plus 384 plate reader using SoftMax Pro v7.0 software.

### Statistical analysis

The number of total negative samples tested for validation was 412, 408, and 436 serum samples for ovine, caprine, and bovine iELISAs, respectively. The number of positive samples used was 27, 29, and 28 samples for ovine, caprine, and bovine iELISAs, respectively. iELISAs were performed in duplicate, with coefficient of variance (COV) analyzed to exclude results that had over 20% COV. The Wilcoxon rank-sum test was performed to analyze the variance between medians of S/P% values between positive and negative serum. Statistical significance was evaluated and noted as ns if *P* > 0.05, * if *P* ≤ 0.05, ** if *P* ≤ 0.02, *** if *P* ≤ 0.001, and **** if *P* ≤ 0.0001. To determine cutoff values, sensitivity, and specificity for iELISAs, receiver operating characteristic (ROC) curves were produced. The area under the curve was also determined to evaluate test efficiency. This was used to determine 95% confidence intervals (CI) for sensitivity and specificity. To visualize the correlation between virus neutralization test (VNT) and iELISA results, a generalized linear model was constructed to plot a line of best fit. Nonparametric analysis of correlation was performed using the Wilcoxon rank sum test. Tukey’s multiple comparison test was used to analyze the significance of differences from using different commercial blocking buffers. All analyses were performed, and graphs were generated using R statistical software (43–45).

## RESULTS

### Consensus sequence analysis

Open reading frames that were aligned were evaluated for similarity based on nucleotide and amino acid sequences. There was a nucleotide sequence similarity of over 98.37%, 98.97%, 96.44%, 97.57%, 97.87%, and 97.36% for consensus sequences against all capripoxvirus strains analyzed for LSDV 60 (L1R), LSDV 74 (p32), LSDV 117 (A27L), LSDV 122 (A33R), LSDV 123 (A34), and LSDV 141 (B5R), respectively (Table S4). Amino acid sequence similarity exceeded 98.37%, 97.21%, 95.3%, 96.44%, 96.49%, and 95.13% for the same strains (Table S5). Following topology analysis of consensus sequences, transmembrane regions and start/stop codons were removed and replaced with restriction sites. The restriction sites added were for NotI (GCGGCCGC) and BamHI (GGATCC) at the 5′ and 3′ ends of the sequence, respectively. *In silico* restriction cloning was performed using Geneious Prime 2021.0.3 to clone expression sequences into the multiple cloning site (MCS) of the vector plasmid prior to submission of sequences for commercial DNA synthesis and cloning into the plasmid.

### Protein expression and purification

**Fig 1:**
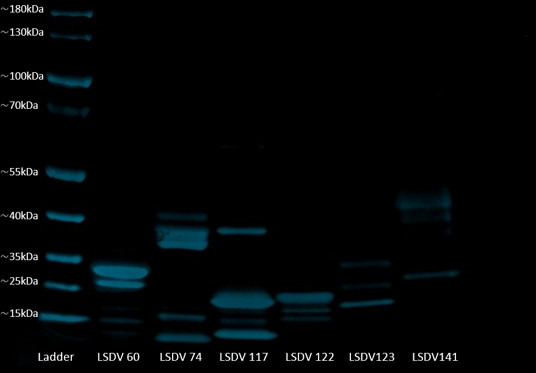
Western blot detection of recombinant LSDV antigens using anti-His antibody. Molecular weights were confirmed using PageRuler prestained protein ladder (ThermoFisher Scientific, catalog number #26616). All proteins were detected at their predicted sizes: LSDV 60 (L1R, 26.85 kDa), LSDV 74 (p32, 37.52 kDa), LSDV 117 (A27L, 17.37 kDa), LSDV 122 (A33R, 22.59 kDa), LSDV 123 (A34, 19.52 kDa), and LSDV 141 (B5R, 26.24 kDa).

Recombinant proteins were successfully produced using a baculovirus expression system in insect cell lines. LSDV 74 (p32), LSDV 123 (A34), and LSDV 141 (B5R) were initially purified using FPLC; however, the proteins precipitated out of solution. Consequently, they were purified using a benchtop protocol that included 10% glycerol in the binding and elution buffers to enhance protein stability. LSDV 60 (L1R), LSDV 117 (A27L), and LSDV 122 (A33R) were purified using FPLC, with no precipitation observed even following multiple freeze-thaw cycles (up to six cycles). The chosen baculovirus vector DNA (ProFold-ER1) allows for the expression of two chaperone proteins, calreticulin and protein disulfide isomerase, which are essential for enhanced protein folding (AB vector). This is critical as the removal of the transmembrane region and intracellular sequences may complicate protein folding. Cloning of the consensus sequences into the plasmid (pAB-Bee-FH) resulted in the fusion of 8× histidine and FLAG tags at the C-terminal end of the expressed proteins. The His tag was used for detection in Western blots with anti-His monoclonal antibody (Fig. 1). Western blotting of proteins showed solid bands at the predicted protein sizes. Faint bands clustered around expected bands are likely due to protein degradation or post-translational modification (46, 47).

### iELISA development and validation

#### iELISA optimization

iELISA development began with sheep serum using checkerboards of various serial dilutions to determine the ideal concentrations of antigen, serum, and conjugate antibody, informed by in-house test development procedures. Subsequent optimizations were conducted in a similar manner using caprine serum followed by bovine serum, informed by initial titrations and analysis with sheep serum. Initial testing using LSDV 74 (p32) and LSDV 141 (B5R) did not result in adequate differentiation between negative and positive serum controls, with negative control absorbance values exceeding 70% of those observed for positive control sera. As a result, the antigens were excluded from further development and validation. Absorbance values ranging from 0.8 to 1.5 were considered suitable for positive control serum to optimize conditions; this range was chosen to capture positive sera of varying strengths while minimizing signal saturation and loss of linearity (48, 49). Nonetheless, a sample-to-positive ratio (S/P%) was used to report iELISA results during validation due to variability in substrate development. Negative serum control showing >40% of the positive control S/P% was considered high background, which led to exclusion of the antigen. For ovine iELISAs, the S/P% of negative control serum was below 35% for all evaluated antigens.

Initial optimization without any blocking agent in the dilution buffer showed that only LSDV 122 (A33R) differentiated between positive and negative controls; the remaining antigens showed high background signal under the same conditions. Several milk and serum-based blocking buffers were initially evaluated, including casein, bovine serum albumin, skim milk, normal chicken serum, normal pig serum, and normal rabbit serum. Skim milk was chosen as the blocking agent due to its accessibility and ability to minimize nonspecific binding of negative serum to the antigen effectively. The buffers used to validate ovine and caprine iELISAs included 5% SMP in PBST for blocking and 2.5% SMP in PBST for serum. Similar serum dilutions were preferentially selected during optimization for comparability of antigen performance and to increase the efficiency to enable multiple tests with the same diluted serum. In most validated iELISAs, serum was diluted 1:25; however, LSDV 123 (A34) iELISAs performed better at a 1:100 serum dilution for both ovine and caprine serum. All antigen iELISAs were validated with caprine serum as the S/P% of negative control serum was below 30% for all optimized tests. Species-specific conjugate antibodies were used for detecting serum to allow for complete evaluation of test efficiency with minimal cross-reactivity. Bovine iELISA development and optimization were more challenging due to the use of skim milk as a dilution buffer. Normal avian and porcine serum, along with skim milk and casein buffer in PBST, were evaluated as potential blocking agents. Only three of the iELISAs were optimized for validation in cattle: LSDV 122 (A33R), LSDV 60 (L1R), and LSDV 117 (A27L), as they were the only tests with acceptable negative control serum S/P% (below 30%).

#### iELISA validation

Over 400 known negative serum samples and over 27 experimentally infected animal serum samples were tested on all optimized iELISAs. All iELISAs were performed in duplicate, with all negative serum validation duplicate sets placed on separate 96-well ELISA plates to increase variability. Standard deviation and coefficient of variation were analyzed to exclude duplicate sets with over 20% variation. S/P% of positive and negative serum were analyzed for variation, and ROC curves were generated to evaluate test efficacy.

#### Ovine iELISA

**Fig 2:**
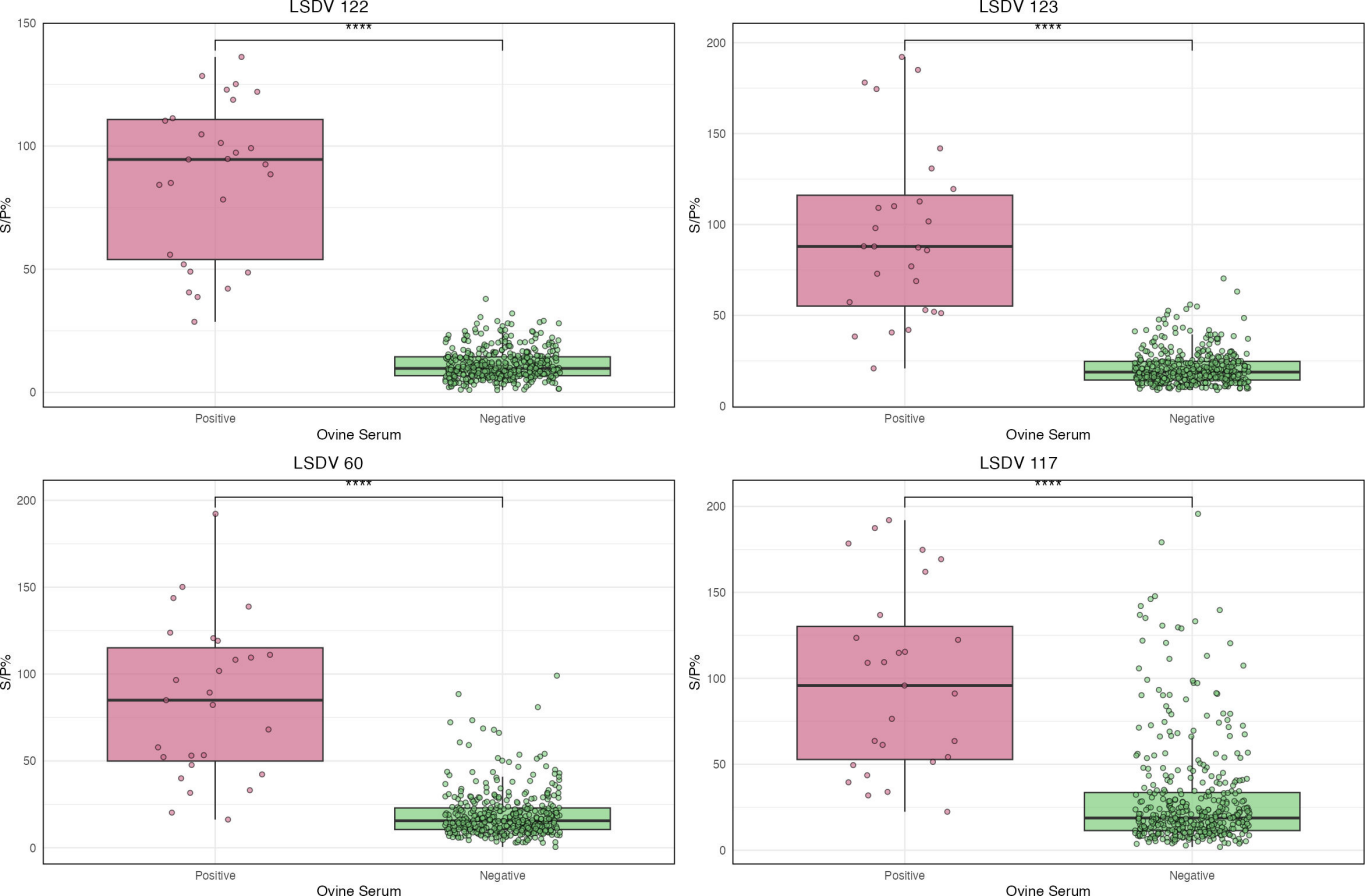
Positive vs negative S/P% comparison of ovine indirect ELISAs with LSDV 122 (A33R), 60 (L1R), 117 (A27L), and 123 (A34) antigens. All antigens showed significant differentiation between groups (Wilcoxon rank-sum test, *P* < 0.0001).

There were 407, 402, and 411 negative serum S/P% values evaluated against 27 positive serum S/P% values in ovine LSDV 122 (A33R), 60 (L1R), and 117 (A27L) iELISAs, respectively (Fig. 2). All tests showed significant variation (*P* < 0.0001) between the two test groups based on nonparametric analysis. Sensitivity and specificity based on ROC curve analysis were determined to be 96.3% (95%CI: 81.72%–99.81%) and 99.26% (95%CI: 98.62%–99.99%) (LSDV 122 A33R), 77.78% (95%CI: 59.24%–89.39%) and 95.27% (95%CI: 92.74%–96.95%) (LSDV 60 L1R), 55.56% (95%CI: 37.31%–72.41%) and 92.21% (95%CI: 89.22%–94.43%) (LSDV 117 A27L), and 92.59% (95%CI: 76.63%–98.68%) and 95.15% (95%CI: 92.62%–96.84%) (LSDV 123 A34). The area under the curve (AUC) for the iELISAs was 0.9995, 0.9503, 0.9041, and 0.9783 with a *P-*value < 0.0001 (Fig. S1). The cutoff S/P% values were 30%, 46.77%, 80%, and 40% for LSDV 122 (A33R), 60 (L1R), 117 (A27L), and 123 (A34) ovine iELISAs, respectively. LSDV 122 and 123 antigens performed exceptionally well for iELISA development with ovine serum, showing relatively high sensitivity and specificity. This is also reflected in the likelihood ratios of the tests, which are 130 and 19 for LSDV 122 (A33R) and 123 (A34) iELISAs, respectively. Although LSDV 60 (L1R) and LSDV 117 (A27L) iELISAs showed good specificity, they were less sensitive. Nonetheless, LSDV 60 (L1R) remains an acceptable diagnostic candidate for ovine serum, with a likelihood ratio of 17.

#### Caprine iELISA

**Fig 3:**
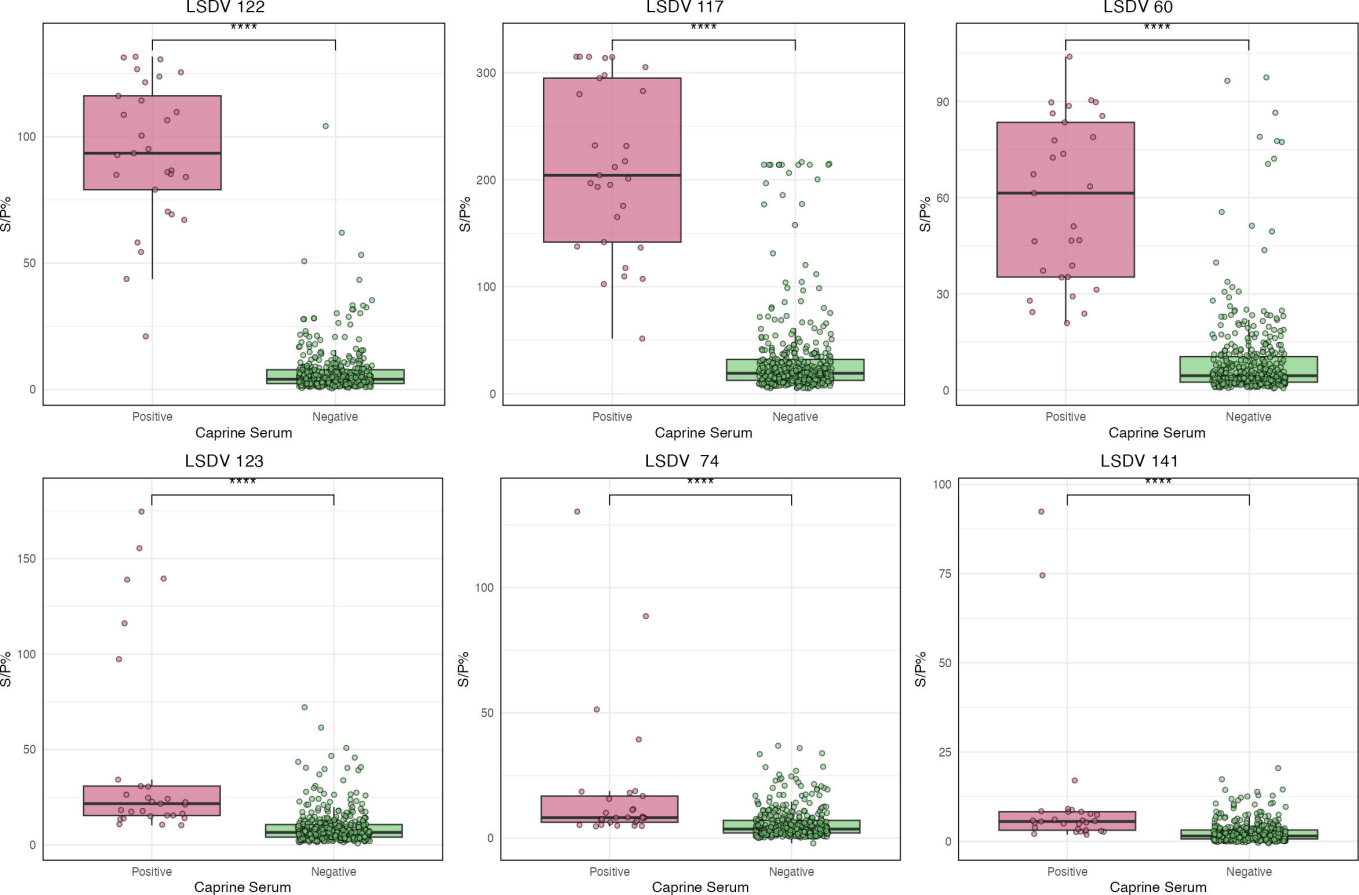
Positive vs negative S/P% comparison of caprine indirect ELISAs developed with LSDV 122 (A33R), 60 (L1R), 117 (A27L), 123 (A34), 141 (B5R), and 74 (P32) antigens. All antigens showed significant differentiation between groups (Wilcoxon rank-sum test, **** P < 0.0001).

There were 404 negative serum S/P% values evaluated against 29 positive serum for LSDV 122 (A33R), 60 (L1R), 123 (A34), 74 (p32), and 141 (B5R) caprine iELISAs and 408 negative serum S/P% values for the LSDV 117 (A27L) caprine iELISA (Fig. 3). All iELISAs showed significant differentiation between positive and negative serum using nonparametric analysis. Sensitivity and specificity were determined to be 93.2% (95%CI: 82.82%–99.82%) and 99.51% (95%CI: 96.80%–99.32%) (LSDV 122 A33R), 78.95% (95%CI: 57.89%–87.78%) and 97.5% (95%CI: 94.57%–98.11%) (LSDV 60 L1R), 96.55% (95%CI: 82.82%–99.82%) and 92% (95%CI: 89.14%–94.39% ) (LSDV 117 A27L), and 31.03% (95%CI: 17.28%–49.23%) and 95% (95%CI: 93.37%–97.36%) (LSDV 123 A34). The likelihood ratios for the iELISAs were 187.1, 23.5, 12.1, and 6.2; the AUC for the iELISAs was 0.9954, 0.9273, 0.8027, and 0.8861 with a *P-*value < 0.0001, respectively (Fig. S2). The cutoff S/P% values for LSDV 122 (A33R), 60 (L1R), 117 (A27L), and 123 (A34) caprine iELISAs were 35%, 30%, 50%, and 30%, respectively. While the LSDV 122 (A33R) iELISA demonstrated excellent diagnostic potential, the LSDV 60 iELISA also showed promising results, confirming its diagnostic value. The LSDV 117 (A27L) iELISA showed adequate specificity but was less sensitive, while LSDV 123 (A34) demonstrated moderate sensitivity and specificity.

#### Bovine iELISA

**Fig 4:**
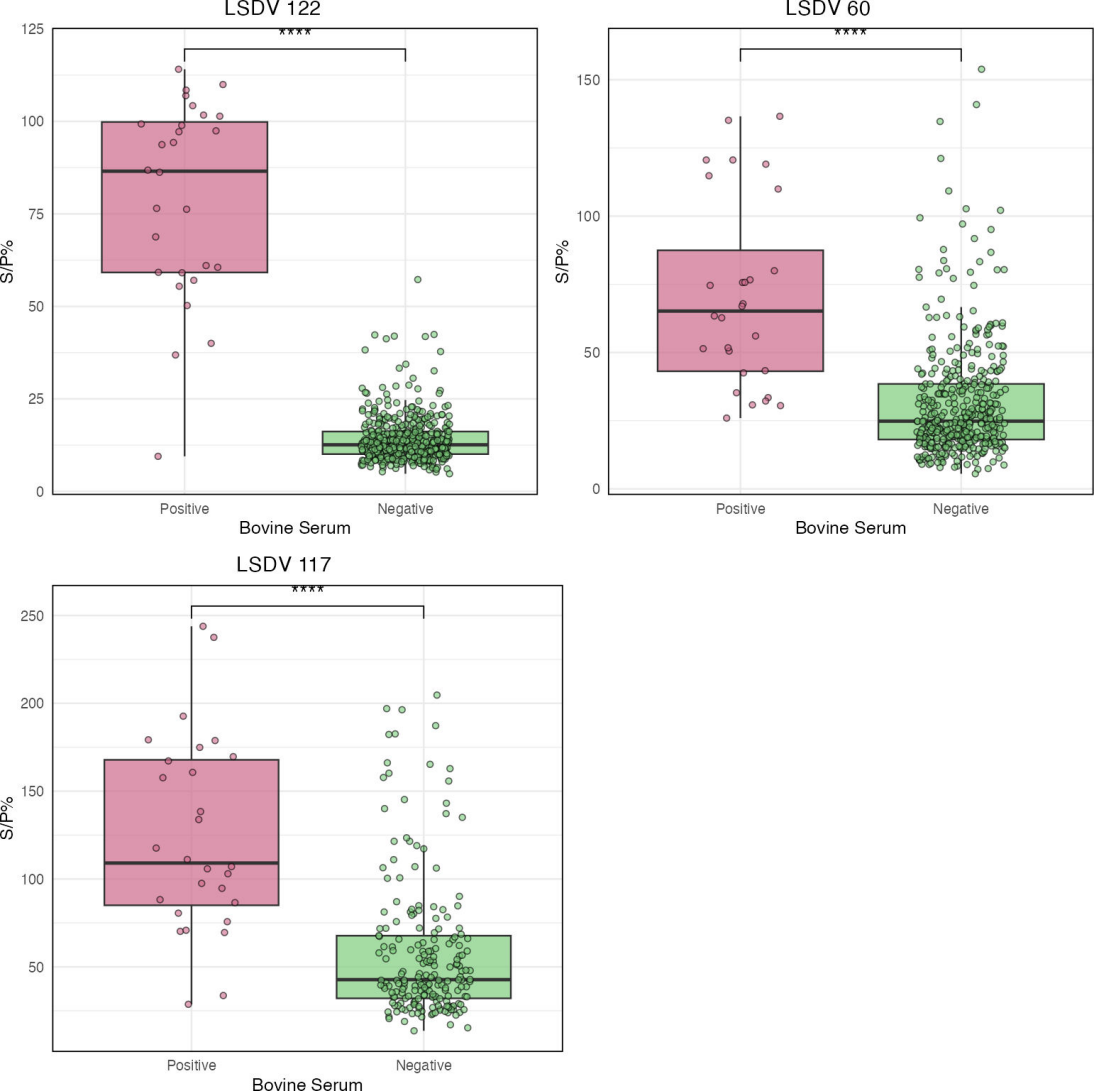
Positive vs negative S/P% comparison of bovine indirect ELISAs developed with LSDV 122 (A33R), 60 (L1R), and 117 (A27L) antigens. All antigens showed significant differentiation between groups (Wilcoxon rank-sum test, *P* < 0.0001).

There were 436, 410, and 201 negative serum S/P% values evaluated against 28 positive serum samples for LSDV 122 (A33R), 60 (L1R), and 117 (A27L) bovine iELISAs (Fig. 4). All iELISAs showed significant differentiation using nonparametric analysis and F-test analysis with *P* < 0.0001. The cutoff S/P% values for bovine iELISAs were determined to be 33.3% for LSDV 122 (A33R), 74.3% for LSDV 60 (L1R), and 77.6% for LSDV 117 (A27L). The AUC for the iELISAs was 0.9701, 0.8677 and 0.8499 for LSDV 122 (A33R), 60 (L1R), and 117 (A27L) (Fig. S3), respectively. Sensitivity and specificity values obtained from the validation of the bovine iELISAs were 96.43% (95%CI: 82.29%–99.82%) and 97.94% (95%CI: 95.83%–98.75%), 42.86% (95%CI: 26.51%–60.93%) and 94.39% (95%CI: 91.44%–96.04%), and 78.57% (95%CI: 60.46%–89.79%) and 79.60% (95%CI: 80.60%–90.18%) for LSDV 122 (A33R), 60 (L1R), and 117 (A27L), respectively. The likelihood ratios were 41.9, 7.2, and 5.9, respectively. These results indicate that both LSDV 122 (A33R) and LSDV 60 (L1R) demonstrate potential for use in diagnostic applications, particularly LSDV 122 (A33R), which has high sensitivity and specificity.

#### Test performance analysis

**Fig 5:**
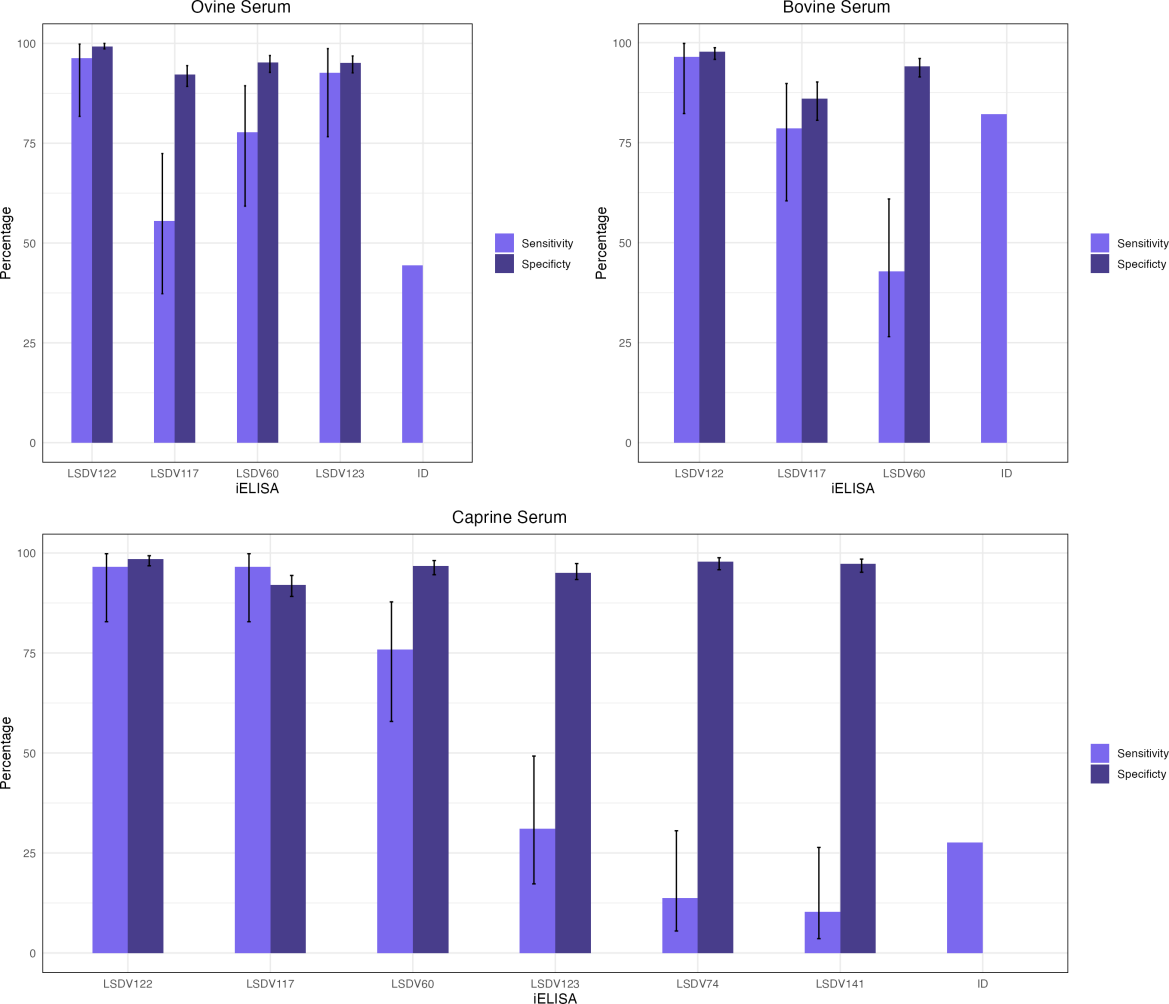
Bar charts of the specificity and sensitivity of validated IELISAs using recombinant antigens in sheep, goat, and cattle sera. Error bars represent 95% confidence intervals. Sensitivity of ID capripoxvirus ELISA was determined using positive samples from IELISA validation.

**Fig 6:**
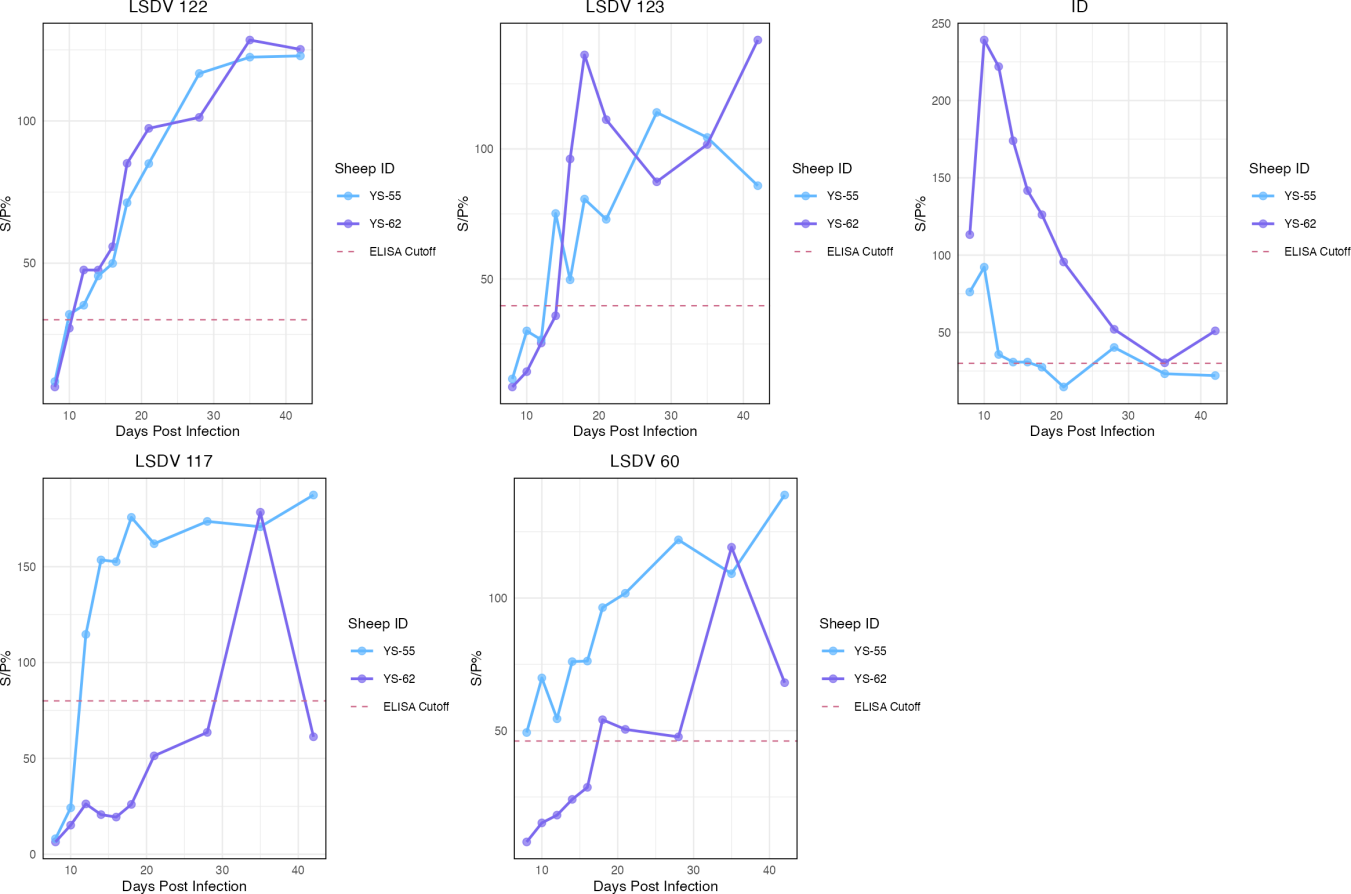
Kinetics of antibody response in two experimentally infected sheep with Yemen goat pox virus. Serum was collected from 8 to 42 days post-infection, and antibody levels were measured using ID ELISA and all ovine iELSAs validated (LSDV 122 [A33R], 60 [L1R], 117 [A27L], and 123 [A34]).

A commercial assay (ID) was used to compare the performance (sensitivity) of iELISAs with known positives used to validate the assays. The ID assay had a sensitivity of 27.59%, 44.44%, and 82.14% for ovine, caprine, and bovine sera, respectively. In caprine sera, the LSDV 122 (A33R), 60 (L1R), and LSDV 117 (A27L) outperformed ID while LSDV 123 (A34) performed similarly (Fig. 5). In ovine sera, LSDV 122 (A33R), LSDV 123 (A34), and LSDV 60 (L1R) iELISAs outperformed the ID assay while LSDV 117 (A27L) performed similarly. In bovine sera, LSDV 122 (A33R) outperformed the ID assay while LSDV 117 (A27L) performed similarly. The ID assay was not analyzed for specificity. The known positives were also analyzed using virus neutralization tests (VNT) to evaluate the correlation of VNT and iELISA results. Scatter plots were generated alongside simple linear regression models to examine if there was an association between VNT results and iELISA results. In caprine sera, a correlation was also observed with the other iELISAs evaluated including the ID assay, excluding LSDV 117 (A27L) (Fig. S4). The Spearman correlation coefficient of LSDV 123 (A34) shows a strong correlation (rs = 0.76, *P* < 0.00001). LSDV 60 (L1R), LSDV 74 (p32), LSDV 122 (A33R), LSDV 141 (B5R), and the ID ELISA had a moderate correlation with VNT results (rs = 0.60, *P* < 0.005; rs = 0,62, *P* < 0.001; rs = 0.61, *P* < 0.005; rs = 0.43, *P* = 0.02; rs = 0.55, *P* = 0.002). This is reflective of the iELISA optimization results in which LSDV 123 (A34) was not found to have a high sensitivity since the positive caprine samples comprised varying neutralizing response. In ovine sera, Spearman’s correlation coefficients showed no significant correlation for LSDV 122 (A33R) and LSDV 60 (L1R), a strong correlation for LSDV 123 (A34) (rs = 0.66, *P* = 0.0002), moderate correlation for the ID ELISA (rs = 0.424, *P* < 0.03), and weak correlation for LSDV 117 (A27L) (rs = 0.39, *P* < 0.05) (Fig. S5). In bovine sera, LSDV 117 (A27L) and LSDV 122 (A34R) did not have any significant correlation using Spearman’s rank test, whereas LSDV 60 (L1R) and the ID ELISA showed a weak correlation with VNT results (rs = 0.39, *P* < 0.04; rs = 0.40, *P* < 0.04) (Fig. S6). Additionally, sera at different time points of two experimentally infected sheep were used to analyze the comparative ability of the LSDV 122 (A33R), LSDV 60 (L1R), LSDV 117 (A27L), and LSDV 123 (A34) iELISAs to detect antibodies compared to the ID assay over time. The assays were able to detect serum as early as DPI 10; furthermore, the S/P% of the iELISAs are reflective of an increase in serum antibody in response to infection (Fig. 6). However, the ID ELISA did not demonstrate an increase in the serum antibody response to infection. Further analysis of the antigens using commercial blocking agents shows the potential for improved performance of antigens that do not perform well using skim milk or casein (Fig. S7).

## DISCUSSION

In this study, soluble baculovirus-expressed proteins LSDV 60 (L1R), LSDV 74 (p32), LSDV 117 (A27L), LSDV 122 (A33R), LSDV 123 (A34), and LSDV 141 (B5R) were evaluated as antigens in indirect ELISAs (iELISA) to detect anti-capripoxvirus antibodies. Antigen selection was informed by the immunogenicity profiles of vaccinia virus homologs in addition to prior studies describing capripoxvirus antigens (29).

Each iELISA was optimized using checkerboard titrations to allow for detection of anti-capripoxvirus antibodies from sheep, goats, and cattle. The commercial ID ELISA was also compared using the same sera used to validate the iELISAs. The results indicated that LSDV 74 (p32) and LSDV 141 (B5R) were not suitable for discriminating between positive and negative sera in goats following optimization and did not discriminate between negative and positive sheep and bovine serum controls. LSDV 74 (p32) has been described as a potential antigen to be used in iELISA by several groups using bacterial (17, 18), yeast (50), and peptide systems (19). Monoclonal antibodies specific to LSDV 74 (p32) have been used to capture inactivated LSDV in indirect (51) and competitive ELISAs (52). One possible reason for the discrepancy is that the positive sera used to evaluate the p32 protein (LSDV 74) were very strong positive sera containing high levels of anti-capripoxvirus antibodies that could be detected in the iELISA. In this study, using baculovirus expressed LSDV 74 (p32), a low percentage of positive sera could be detected, while a correlation between the LSDV 74 (p32) iELISA against VNT results from positive caprine serum was observed. Another possible reason for the contradictory results is the use of a consensus sequence based on all three capripoxviruses; this may have resulted in epitope modifications affecting the specificity of the antigenic epitopes on LSDV 74 (p32). Prior studies on the use of LSDV 74 (p32) to develop serological assays have utilized a native virus sequence for protein production. Protein misfolding is not the likely cause as the LSDV 74 (p32) antigen was effective in iELISAs using mice and rabbit sera exposed to LSDV 74 (p32) antigen, with sufficient discrimination between positive and negative sera (manuscript in preparation). Based on the findings, this antigen as a soluble antigen does not seem to be suitable for a diagnostic capripoxvirus ELISA.

The commercial ID ELISA has a claim of very high specificity in capripoxvirus-free regions (>99.7%) and at least equivalent sensitivity compared to IPMA and improved sensitivity compared to VNT. The ID ELISA performed reasonably well in detecting anti-capripoxvirus antibodies in cattle sera used in this study with a sensitivity around 80%, although it missed several samples that were positive by VNT. This commercial ELISA has been used in several studies (13, 14, 53–58). However, the stated sensitivity of the test is higher than was observed with this study. A study in Thailand had similar findings demonstrating that the VNT detected a higher number of cattle compared to the ID ELISA (55). The implications of this are that these studies likely underestimated the presence of capripoxvirus specific antibodies in the populations evaluated. Furthermore, the ID ELISA did not perform well in detecting anti-capripoxvirus antibodies with the sheep and goat sera used in this study. This result agrees with that of another study that determined that the commercial ELISA showed poor sensitivity for SPPV compared to VNT (59) and that the commercial ELISA detected antibodies much later in LSDV-infected cattle (11). In addition, using serum collected at different time points from sheep experimentally infected with Yemen GTPV virus did not demonstrate increasing antibodies with time using the ID ELISA, as observed with proteins: LSDV 60 (L1R), LSDV 117 (A27L), LSDV 122 (A33R), and LSDV 123 (A34). The ID ELISA showed low specificity at early time points and missed detecting antibody response at later time points. These sheep had very mild clinical disease and low levels of capripoxvirus antibodies, which is a likely reason why the ID ELISA was not able to detect these samples.

LSDV 60 (L1R) was able to detect anti-capripoxvirus antibodies with a sensitivity and specificity of 75.86% and 96.78% in goats, 77.78% and 95.27% in sheep, and 42.86% and 94.39% in cattle, respectively. LSDV 60 (L1R) was demonstrated as a potential antigen for a lumpy skin disease iELISA using a small number of sera (20). Evaluation of LSDV 60 (L1R) for use in a competitive ELISA would likely reduce the background from nonspecific binding and improve assay sensitivity due to increased specificity associated with the use of a monoclonal antibody (60).

LSDV 117 (A27L) was able to detect anti-capripoxvirus antibodies with a sensitivity and specificity of 96.55% and 92.16% in goats, 55.56% and 92.21% in sheep, and 78.57% and 79.60% in cattle, respectively. LSDV 117 (A27L) was previously expressed using *E. coli* and evaluated using sera from capripoxvirus-infected sheep and goats (21). More recently, it has been validated for use in iELISA for sheep pox and goat pox in India (22). Both studies, which used serum neutralization tests as a reference, show that 117 (A27L) can be a potential antigen used for diagnostics for detecting anti-capripoxvirus antibodies in sheep and goats, further demonstrating its potential use in cattle.

LSDV 122 (A33R) was demonstrated overall to be the most sensitive and specific antigen for detecting anti-capripoxvirus antibodies in sheep, goats, and cattle with sensitivity and specificity of 96.3% and 99.26%, 96.55% and 98.5%, and 96.43% and 97.94%, respectively. LSDV 122 (A33R) has been recently reported as an effective capripoxvirus ELISA in sheep and goats (23) and cattle using an indirect (24) and competitive ELISA (25). The nonsignificant correlation of LSDV 122 (A33R) with VNT results in sheep and cattle sera further highlights its diagnostic capability to capture serum response that is not detected using VNTs. Since LSDV 122 (A33R) is an extracellular enveloped virus (EEV) protein, it is not unexpected that it does not correlate with VNT results as VNT primarily measures neutralization of intracellular mature virus (IMV).

LSDV 123 (A34) was demonstrated to be a potentially useful antigen for detecting capripoxvirus-specific antibodies in sheep and goats with sensitivity and specificity of 92.59% and 95.15% and 78.95% and 90.64%, respectively. This aligns with the previously reported results (12). The strong correlation of LSDV 123 (A34) iELISAs and VNT results reflects its enhanced performance when validated using VNT-positive serum samples.

Although there are no serotypes among capripoxvirus, there are subtle species-specific differences with respect to the immune responses generated. It was demonstrated that an immunoperoxidase monolayer assay (IPMA) used LSDV as the antigen is more sensitive for detecting antibodies to LSDV compared to sheep pox or goat pox (11). In addition, recently, it was demonstrated that there were differences observed in VNT between using homologous virus or heterologous virus (61). This study, which compared sera collected from sheep, goats, and cattle experimentally infected with different capripoxviruses, demonstrated there are differences in the populations with respect to the immune responses generated to different antigens. This is reflected by the varying sensitivities of the antigens evaluated and further demonstrates that although there are no serotypes between capripoxviruses, there are differences in the immune responses generated between these viruses and their hosts. However, LSDV 122 (A33R) was highly sensitive and specific in sheep, goats, and cattle, making it an ideal candidate for a single antigen capripoxvirus ELISA.

Continued development and evaluation of serological tests for capripoxviruses are necessary for improved control of SPPV, GTPV, and LSDV. This study demonstrated that several different antigens can be used to detect anti-capripoxvirus antibodies in an iELISA. Identifying different antigens as diagnostic targets can allow for enhanced diagnosis of capripoxvirus infection. This can be accomplished by testing sera on two or more different iELISAs or alternatively by using different antigens on platforms such as Luminex (62). The pairwise comparison of LSDV antigens using accessible reagents resulted in varying sensitivity and specificities, with some iELISAs having unusually high cutoff values dependent on antigen and animal species. Additional evaluation of antigen performance using commercial blocking buffers showed improved performance in some antigens. This highlights the need for systematic evaluation of antigen performance under multiple conditions.

Since the antigens used are recombinant proteins, they are safe and can be used in lower containment facilities. Further work to improve the iELISAs and commercialize the tests with further validation will allow for better serological diagnostics for SPPV, GTPV, and LSDV. In addition, the development of monoclonal antibodies to allow for blocking or competitive ELISAs could also improve the sensitivity and specificity of the diagnostic tests.

The development and validation of iELISAs utilizing recombinant LSDV antigens demonstrates robust capabilities for detecting capripoxvirus infections. The sensitivity and specificity metrics achieved suggest that these assays can significantly contribute to the monitoring and control of capripoxviruses in endemic areas where access to commercial tests may be limited. Future work should focus on further refining these assays for validation studies using field sera as it has been observed that with capripoxvirus ELISAs based on core proteins 095 and 103 performed well with sera from experimentally infected animals, but this performance was not observed using field sera. In addition, the integration of improved ELISAs into broader surveillance programs to manage capripoxvirus outbreaks effectively is being pursued.

## SUPPLEMENTAL MATERIAL

Supplemental tables and figures jcm.01191-25-s0001.pdf Tables S1 to S5 and Figures S1 to S7.

ASM does not own the copyrights to Supplemental Material that may be linked to, or accessed through, an article. The authors have granted ASM a non-exclusive, world-wide license to publish the Supplemental Material files. Please contact the corresponding author directly for reuse.

## References

[1] Tulman ER, Afonso CL, Lu Z, Zsak L, Sur J-H, Sandybaev NT, Kerembekova UZ, Zaitsev VL, Kutish GF, Rock DL. 2002. The genomes of sheeppox and goatpox viruses. J Virol 76:6054–6061. doi:10.1128/jvi.76.12.6054-6061.2002 12021338 PMC136203

[2] Tuppurainen ESM, Venter EH, Shisler JL, Gari G, Mekonnen GA, Juleff N, Lyons NA, De Clercq K, Upton C, Bowden TR, et al.. 2017. Review: capripoxvirus diseases: current status and opportunities for control. Transbound Emerg Dis 64:729–745. doi:10.1111/tbed.12444 26564428 PMC5434826

[3] Babiuk Shawn, Bowden TR, Parkyn G, Dalman B, Hoa DM, Long NT, Vu PP, Bieu DX, Copps J, Boyle DB. 2009. Yemen and Vietnam capripoxviruses demonstrate a distinct host preference for goats compared with sheep. J Gen Virol 90:105–114. doi:10.1099/vir.0.004507-0 19088279

[4] Babiuk S, Bowden TR, Boyle DB, Wallace DB, Kitching RP. 2008. Capripoxviruses: an emerging worldwide threat to sheep, goats and cattle. Transbound Emerg Dis 55:263–272. doi:10.1111/j.1865-1682.2008.01043.x 18774991

[5] Mazloum A, Van Schalkwyk A, Babiuk S, Venter E, Wallace DB, Sprygin A. 2023. Lumpy skin disease: history, current understanding and research gaps in the context of recent geographic expansion. Front Microbiol 14:1266759. doi:10.3389/fmicb.2023.1266759 38029115 PMC10652407

[6] Villalba R, Haegeman A, Ruano MJ, Gómez MB, Cano-Gómez C, López-Herranz A, Tejero-Cavero J, Capilla J, Bascuñan MV, De Regge N, et al.. 2024. Lessons learned from active clinical and laboratory surveillance during the sheep pox virus outbreak in Spain, 2022–2023. Viruses 16:1034. doi:10.3390/v16071034 39066197 PMC11281627

[7] World Organisation for Animal Health. 2026. WAHIS [Internet]. Available from: https://wahis.woah.org/#/home

[8] Nugroho W, Mardani HM, Reichel MP, Fitria Y, Miswati Y, Febrianto N, Nuryanto ME, Apriana I, Azzahrawani N, Martalina E, et al.. 2024. The first outbreak of lumpy skin disease in Indonesia. Trop Anim Health Prod 56:237. doi:10.1007/s11250-024-04067-y 39110359

[9] Badara O. 2025. Statement on recent lumpy skin disease outbreaks in Europe [Internet]. WOAH - World Organisation for Animal Health. Available from: https://www.woah.org/en/statement-on-recent-lumpy-skin-disease-outbreaks-in-europe/

[10] Yeşilbağ K, Toker EB, Yaşar M, Casal J, Pratelli A. 2026. Lumpy skin disease threat in Europe: current situation, transmission dynamics and future prospects. Res Vet Sci 202:106061. doi:10.1016/j.rvsc.2026.106061 41548460

[11] Haegeman A, De Leeuw I, Mostin L, Van Campe W, Aerts L, Vastag M, De Clercq K. 2020. An immunoperoxidase monolayer assay (IPMA) for the detection of lumpy skin disease antibodies. J Virol Methods 277:113800. doi:10.1016/j.jviromet.2019.113800 31837373 PMC6996284

[12] Berguido FJ, Gelaye E, Liu Y, Davaasuren B, Krstevski K, Djadjovski I, Ivanova E, Goujgoulova G, Loitsch A, Tuppurainen E, et al.. 2022. Development and optimization of indirect ELISAs for the detection of anti-capripoxvirus antibodies in cattle, sheep, and goat sera. Microorganisms 10:1956. doi:10.3390/microorganisms10101956 36296232 PMC9608586

[13] Krešić N, Šimić I, Bedeković T, Acinger-Rogić Ž, Lojkić I. 2020. Evaluation of serological tests for detection of antibodies against lumpy skin disease virus. J Clin Microbiol 58:e00348-20. doi:10.1128/JCM.00348-20 32434783 PMC7448653

[14] Milovanović M, Milićević V, Radojičić S, Valčić M, Hoffmann B, Dietze K. 2020. Suitability of individual and bulk milk samples to investigate the humoral immune response to lumpy skin disease vaccination by ELISA. Virol J 17:28. doi:10.1186/s12985-020-01298-x 32138740 PMC7059690

[15] Babiuk S, Wallace DB, Smith SJ, Bowden TR, Dalman B, Parkyn G, Copps J, Boyle DB. 2009. Detection of antibodies against capripoxviruses using an inactivated sheeppox virus ELISA. Transbound Emerg Dis 56:132–141. doi:10.1111/j.1865-1682.2009.01067.x 19281604

[16] Bowden TR, Coupar BE, Babiuk SL, White JR, Boyd V, Duch CJ, Shiell BJ, Ueda N, Parkyn GR, Copps JS, et al.. 2009. Detection of antibodies specific for sheeppox and goatpox viruses using recombinant capripoxvirus antigens in an indirect enzyme-linked immunosorbent assay. J Virol Methods 161:19–29. doi:10.1016/j.jviromet.2009.04.031 19426763

[17] Heine HG, Stevens MP, Foord AJ, Boyle DB. 1999. A capripoxvirus detection PCR and antibody ELISA based on the major antigen P32, the homolog of the vaccinia virus H3L gene. J Immunol Methods 227:187–196. doi:10.1016/s0022-1759(99)00072-1 10485266

[18] Venkatesan G, Kumar Teli M, Sankar M, Kumar A, Dashprakash M, Arya S, Madhavan A, Ramakrisnan MA, Pandey AB. 2018. Expression and evaluation of recombinant P32 protein based ELISA for sero-diagnostic potential of capripox in sheep and goats. Mol Cell Probes 37:48–54. doi:10.1016/j.mcp.2017.11.005 29158139

[19] Tian H, Chen Y, Wu J, Shang Y, Liu X. 2010. Serodiagnosis of sheeppox and goatpox using an indirect ELISA based on synthetic peptide targeting for the major antigen P32. Virol J 7:245. doi:10.1186/1743-422X-7-245 20854693 PMC2949846

[20] Ntombela N, Matsiela M, Zuma S, Hiralal S, Naicker L, Mokoena N, Khoza T. 2023. Production of recombinant lumpy skin disease virus A27L and L1R proteins for application in diagnostics and vaccine development. Vaccine X 15:100384. doi:10.1016/j.jvacx.2023.100384 37736535 PMC10509699

[21] Dashprakash M, Venkatesan G, Kumar A, Sankar M, Arya S, Ramakrishnan MA, Pandey AB, Mondal B. 2019. Prokaryotic expression, purification and evaluation of goatpox virus ORF117 protein as a diagnostic antigen in indirect ELISA to detect goatpox. Arch Virol 164:1049–1058. doi:10.1007/s00705-019-04170-8 30778744

[22] Manjunatha Reddy GB, Sudeep N, Apsana R, Sumana K, Sai Mounica P, Yogisharadhya R, Balamurugan V, Rajeswari S, Sathish SB. 2025. Development and validation of recombinant A27L based indirect-ELISA for Sheeppox and Goatpox disease in India. Vet Res Commun 49:90. doi:10.1007/s11259-025-10656-5 39869249

[23] Kushwaha A, Kumar A, Chandrasekhar S, Poulinlu G, Chand K, Muthuchelvan D, Venkatesan G. 2024. Baculovirus expression and purification of virion core and envelope proteins of goatpox virus to evaluate their diagnostic potential. Arch Virol 169:172. doi:10.1007/s00705-024-06079-3 39096433

[24] Angsujinda K, Kitchanakan P, Daewang N, Chintapitaksakul L, Wanganurakkul S, Chaiyo S, Khongchareonporn N, Mahony TJ, Assavalapsakul W. 2025. Evaluation of recombinant extracellular enveloped virion protein candidates for the detection of serological responses to lumpy skin disease virus in cattle. Vet Q 45:1–13. doi:10.1080/01652176.2025.2475989 PMC11924265 40103407

[25] Chen G, He X, Gao Z, Fang Y, Hurisa TT, Jia H, Tan J, Zhou G, Fu B, Li W, et al.. 2024. Development of a competitive ELISA based on the LSDV A33 antigen. Virol J 21:203. doi:10.1186/s12985-024-02448-1 39198861 PMC11360308

[26] Wang W, Shi Z, Luo J, Liao H, Feng L, Zhu Y, Lin Y, Shi X, Zhang F, Xi T, et al.. 2025. Development of a double-antigen sandwich ELISA for rapid and accurate detection of antibodies against Capripoxvirus. Microbiol Spectr 13:e0272924. doi:10.1128/spectrum.02729-24 40323098 PMC12131728

[27] Berguido FJ, Chibssa TR, Loitsch A, Liu Y, Krstevski K, Djadjovski I, Tuppurainen E, Petrović T, Vidanović D, Caufour P, et al.. 2023. Harnessing attenuation-related mutations of viral genomes: development of a serological assay to differentiate between capripoxvirus-infected and -vaccinated animals. Viruses 15:2318. doi:10.3390/v15122318 38140559 PMC10747038

[28] Rittipornlertrak A, Modethed W, Sangkakam K, Muenthaisong A, Vinitchaikul P, Boonsri K, Pringproa K, Punyapornwithaya V, Kreausukon K, Sthitmatee N, et al.. 2024. Persistence of passive immunity in calves receiving colostrum from cows vaccinated with a live attenuated lumpy skin disease vaccine and the performance of serological tests. Front Vet Sci 11:1303424. doi:10.3389/fvets.2024.1303424 38835894 PMC11148353

[29] Teffera M, Boshra H, Bowden TR, Babiuk S. 2025. Which proteins? the challenge of identifying the protective antigens for next-generation capripoxvirus vaccines. Vaccines (Basel) 13:219. doi:10.3390/vaccines13030219 40266091 PMC11946534

[30] Sternke M, Tripp KW, Barrick D. 2020. The use of consensus sequence information to engineer stability and activity in proteins. Methods Enzymol 643:149–179. doi:10.1016/bs.mie.2020.06.001 32896279 PMC8098710

[31] Sun PD, Foster CE, Boyington JC. 2004. Overview of protein structural and functional folds. Curr Protoc Protein Sci Chapter 17:Unit. doi:10.1002/0471140864.ps1701s35 PMC7162418 18429251

[32] Munson M, Balasubramanian S, Fleming KG, Nagi AD, O’Brien R, Sturtevant JM, Regan L. 1996. What makes a protein a protein? hydrophobic core designs that specify stability and structural properties. Protein Sci 5:1584–1593. doi:10.1002/pro.5560050813 8844848 PMC2143493

[33] Thermo Fisher Scientific. 2026. Western Blot Sample Preparation Protocol - CA. Available from: https://www.thermofisher.com/ca/en/home/life-science/protein-biology/protein-biology-learning-center/protein-gel-electrophoresis-information/western-blot-protocols/western-blot-sample-preparation.html

[34] Thermo Fisher Scientific. 2026. Thermo Scientific Nanodrop Spectrophotometers. Available from: https://documents.thermofisher.com/TFS-Assets/CAD/Warranties/Thermo-Scientific-NanoDrop-Products-Protein-Technical-Guide-EN.pdf

[35] Block H, Maertens B, Spriestersbach A, Brinker N, Kubicek J, Fabis R, Labahn J, Schäfer F. 2009. Chapter 27, Immobilized-metal affinity chromatography (IMAC): a review, p 439–473. *In* Methods in enzymology. Vol. Available from. Academic Press. 10.1016/S0076-6879(09)63027-5 19892187

[36] Bornhorst JA, Falke JJ. 2000. Purification of proteins using polyhistidine affinity tags. Methods Enzymol 326:245–254. doi:10.1016/s0076-6879(00)26058-8 11036646 PMC2909483

[37] Babiuk S, Bowden TR, Parkyn G, Dalman B, Manning L, Neufeld J, Embury-Hyatt C, Copps J, Boyle DB. 2008. Quantification of lumpy skin disease virus following experimental infection in cattle. Transbound Emerg Dis 55:299–307. doi:10.1111/j.1865-1682.2008.01024.x 18503511

[38] Bamouh Z, Elarkam A, Elmejdoub S, Hamdi J, Boumart Z, Smith G, Suderman M, Teffera M, Wesonga H, Wilson S, et al.. 2024. Evaluation of a combined live attenuated vaccine against lumpy skin disease, contagious bovine pleuropneumonia and rift valley fever. Vaccines (Basel) 12:302. doi:10.3390/vaccines12030302 38543936 PMC10975446

[39] Bowden TR, Babiuk SL, Parkyn GR, Copps JS, Boyle DB. 2008. Capripoxvirus tissue tropism and shedding: a quantitative study in experimentally infected sheep and goats. Virology (Auckl) 371:380–393. doi:10.1016/j.virol.2007.10.002 PMC9955785 17988703

[40] Boshra H, Truong T, Babiuk S, Pox G. 2015. Peste des petits ruminants and rift valley fever in Saudi Arabia. PLoS One 10:e0140328. doi:10.1371/journal.pone.0140328 26462199 PMC4604144

[41] Wolff J, Beer M, Hoffmann B. 2020. Thermal inactivation of different capripox virus isolates. Microorganisms 8:2053. doi:10.3390/microorganisms8122053 33371463 PMC7767500

[42] Jones FS. 1927. The effect of heat on antibodies. J Exp Med 46:291–301. doi:10.1084/jem.46.2.291 19869339 PMC2131163

[43] The R Core Team. 2025. R: a language and environment for statistical computing. R Foundation for Statistical Computing. https://cran.r-project.org/doc/manuals/r-release/fullrefman.pdf.

[44] Wickham H. 2009. Ggplot2: elegant graphics for data analysis. Springer New York, New York, NY. https://link.springer.com/10.1007/978-0-387-98141-3.

[45] Robin X, Turck N, Hainard A, Tiberti N, Lisacek F, Sanchez J-C, Müller M. 2011. pROC: an open-source package for R and S+ to analyze and compare ROC curves. BMC Bioinformatics 12:77. doi:10.1186/1471-2105-12-77 21414208 PMC3068975

[46] Pillai-Kastoori L, Heaton S, Shiflett SD, Roberts AC, Solache A, Schutz-Geschwender AR. 2020. Antibody validation for Western blot: By the user, for the user. J Biol Chem 295:926–939. doi:10.1074/jbc.RA119.010472 31819006 PMC6983856

[47] Chen N, Kong X, Zhao S, Xiaofeng W. 2020. Post-translational modification of baculovirus-encoded proteins. Virus Res 279:197865. doi:10.1016/j.virusres.2020.197865 31987850

[48] Alhajj M, Zubair M, Farhana A. 2025. Enzyme linked immunosorbent assay. *In* StatPearls. Treasure Island (FL): StatPearls Publishing. 32310382

[49] Aydin S, Emre E, Ugur K, Aydin MA, Sahin İ, Cinar V, Akbulut T. 2025. An overview of ELISA: a review and update on best laboratory practices for quantifying peptides and proteins in biological fluids. J Int Med Res 53:03000605251315913. doi:10.1177/03000605251315913 39922798 PMC11808753

[50] Bhanot V, Balamurugan V, Bhanuprakash V, Venkatesan G, Sen A, Yadav V, Yogisharadhya R, Singh RK. 2009. Expression of P32 protein of goatpox virus in Pichia pastoris and its potential use as a diagnostic antigen in ELISA. J Virol Methods 162:251–257. doi:10.1016/j.jviromet.2009.08.020 19733197

[51] Baselli S, Pezzoni G, Sabino M, Grazioli S, Wolff J, Hoffmann B, Shtjefni V, Capucci L, Brocchi E. 2023. ELISA methods based on monoclonal antibodies for the serological diagnosis of lumpy skin disease. Transbound Emerg Dis 2023:8378153. doi:10.1155/2023/8378153 40303676 PMC12017155

[52] Baselli S, Hoffmann B, Milovanović M, Shtjefni V, Ricchi M, Sabino M, Grazioli S, Brocchi E, Pezzoni G. 2025. Enhancing lumpy skin disease control: effective competitive and indirect ELISAs for serological surveillance. J Virol Methods 333:115108. doi:10.1016/j.jviromet.2025.115108 39793900

[53] Milovanović M, Dietze K, Milićević V, Radojičić S, Valčić M, Moritz T, Hoffmann B. 2019. Humoral immune response to repeated lumpy skin disease virus vaccination and performance of serological tests. BMC Vet Res 15:80. doi:10.1186/s12917-019-1831-y 30841894 PMC6404298

[54] Ochwo S, VanderWaal K, Munsey A, Nkamwesiga J, Ndekezi C, Auma E, Mwiine FN. 2019. Seroprevalence and risk factors for lumpy skin disease virus seropositivity in cattle in Uganda. BMC Vet Res 15:236. doi:10.1186/s12917-019-1983-9 31286926 PMC6615106

[55] Suwankitwat N, Bhakha K, Molee L, Songkasupa T, Puangjinda K, Chamchoy T, Arjkumpa O, Nuansrichay B, Srisomrun S, Pongphitcha P, et al.. 2023. Long-term monitoring of immune response to recombinant lumpy skin disease virus in dairy cattle from small-household farms in western Thailand. Comp Immunol Microbiol Infect Dis 99:102008. doi:10.1016/j.cimid.2023.102008 37467568

[56] Sareyyüpoğlu B, Uzar S, Saraç F, Enül H, Adıay C, Çokçalışkan C, Arslan A, Öztap G, Gülyaz V. 2023. Immune response against lumpy skin disease after simultaneous vaccination of cattle with sheep pox and goat pox and foot and mouth disease vaccines. Vet Microbiol 281:109726. doi:10.1016/j.vetmic.2023.109726 37054661

[57] Hakobyan V, Sargsyan K, Elbakyan H, Sargsyan V, Markosyan T, Chobanyan G, Badalyan M, Kharatyan S. 2024. Duration of immunity in cattle to lumpy skin disease utilizing a sheep pox vaccine. Vet Sci 11:164. doi:10.3390/vetsci11040164 38668431 PMC11053425

[58] Parvin R, Al Mim S, Haque MN, Jerin I, Nooruzzaman M, Hossain MR, Chowdhury EH, Globig A, Knauf S, Tuppurainen E. 2025. Serological response to lumpy skin disease in recovered and clinically healthy vaccinated and unvaccinated cattle of Bangladesh. Front Vet Sci 12:1535600. doi:10.3389/fvets.2025.1535600 40034563 PMC11873106

[59] Rhazi H, Mikou K, Sadeqy Y, Alhayane M, El Mejdoub S, Safini N, Lenk M, Tadlaoui KO, Elharrak M. 2022. Evaluation of ELISA and VNT for sheeppox virus antibody detection and development of an immunoenzymatic quantitative method. J Immunol Methods 502:113226. doi:10.1016/j.jim.2022.113226 35032520

[60] Hayrapetyan H, Tran T, Tellez-Corrales E, Madiraju C. 2023. Enzyme-linked immunosorbent assay: types and applications, p 1–17. *In* Matson RS (ed), ELISA: methods and protocols [Internet]. Vol. Available from. Springer US, New York, NY. 10.1007/978-1-0716-2903-1_1 36795355

[61] Berguido FJ, Kangethe RT, Shell W, Wijewardana V, Grabherr R, Cattoli G, Lamien CE. 2024. Different neutralizing antibody responses of heterologous sera on sheeppox and lumpy skin disease viruses. Viruses 16:1127. doi:10.3390/v16071127 39066289 PMC11281510

[62] Berguido FJ, Settypalli TBK, Mbuyi CGT, Bakhom MT, van Vuren PJ, Pawęska JT, Cattoli G, Grabherr R, Lamien CE. 2024. Development of a luminex-based assay for the detection of anti-capripoxvirus and rift valley fever virus antibodies in domestic ruminants. Virol J 21:335. doi:10.1186/s12985-024-02602-9 39726039 PMC11674245

