# Supplementary material for: Comparison of multiple antigens for use in indirect ELISAs for detection of anti-capripoxvirus antibodies in sheep, goats, and cattle
Source: J Clin Microbiol. 2026 Jun 12;64(7):e01191-25. doi: 10.1128/jcm.01191-25 (PMC13343892; doi:10.1128/jcm.01191-25)
Supplement: Supplemental tables and figures — Tables S1 to S5 and Figures S1 to S7. [file jcm.01191-25-s0001.pdf]

Supplementary Table 1. Statistical analysis of commercial blocking buffer comparisons with skim milk for IELISAs using positive and negative caprine serum.

	Tukey's multiple comparisons test (Caprine)	Mean Diff.	95.00% CI of diff.	Summary	Adjusted P Value
**Skim Milk**	122-Pos vs. 122-Neg	1.768	0.8197 to 2.717	*	0.0211
	60-Pos vs. 60-Neg	1.822	1.167 to 2.478	*	0.0211
	117-Pos vs. 117-Neg	1.02	-0.8703 to 2.911	ns	0.1018
	123-Pos vs. 123-Neg	0.883	0.3962 to 1.370	*	0.0211
	141-Pos vs. 141-Neg	0.8469	0.7179 to 0.9759	*	0.0211
	74-Pos vs. 74-Neg	0.726	0.4281 to 1.024	*	0.0211
**Super block**	122-Pos vs. 122-Neg	1.734	1.680 to 1.788	*	0.0211
	60-Pos vs. 60-Neg	1.852	1.747 to 1.957	*	0.0211
	117-Pos vs. 117-Neg	0.9188	-0.2654 to 2.103	ns	0.0784
	123-Pos vs. 123-Neg	1.004	0.8995 to 1.109	*	0.0211
	141-Pos vs. 141-Neg	1.058	-0.5893 to 2.704	ns	0.0911
	74-Pos vs. 74-Neg	0.7647	0.4168 to 1.112	*	0.0211
**Starting block**	122-Pos vs. 122-Neg	1.8	1.140 to 2.460	*	0.0211
	60-Pos vs. 60-Neg	1.867	1.005 to 2.730	*	0.0211
	117-Pos vs. 117-Neg	0.7676	-1.855 to 3.390	ns	0.1867
	123-Pos vs. 123-Neg	0.831	-0.1510 to 1.813	ns	0.0699
	141-Pos vs. 141-Neg	0.9433	0.3890 to 1.497	*	0.0212
	74-Pos vs. 74-Neg	0.7379	0.1962 to 1.280	*	0.0244
**Assay buffer**	122-Pos vs. 122-Neg	1.47	0.1818 to 2.758	*	0.0355
	60-Pos vs. 60-Neg	1.724	1.654 to 1.794	*	0.0211
	117-Pos vs. 117-Neg	0.9647	0.8981 to 1.031	*	0.0211
	123-Pos vs. 123-Neg	0.8962	0.5043 to 1.288	*	0.0211
	141-Pos vs. 141-Neg	1.051	-0.09159 to 2.194	ns	0.0603
	74-Pos vs. 74-Neg	0.8622	0.3479 to 1.376	*	0.0213
**Pierce buffer**	122-Pos vs. 122-Neg	1.556	0.8819 to 2.230	*	0.0211
	60-Pos vs. 60-Neg	1.699	0.08157 to 3.317	*	0.0442
	117-Pos vs. 117-Neg	0.9524	0.8908 to 1.014	*	0.0211
	123-Pos vs. 123-Neg	0.7988	0.2305 to 1.367	*	0.0235
	141-Pos vs. 141-Neg	1.099	-0.1824 to 2.381	ns	0.0684
	74-Pos vs. 74-Neg	0.8046	0.6307 to 0.9785	*	0.0211

Supplementary Table 2. Statistical analysis of commercial blocking buffer comparisons with skim milk for IELISAs using positive and negative ovine serum.

	Tukey's multiple comparisons test (Ovine)	Mean Diff.	95.00% CI of diff.	Summary	Adjusted P Value
**Skim Milk**	122-Pos vs. 122-Neg	1.892	1.048 to 2.736	*	0.0211
	60-Pos vs. 60-Neg	1.02	-0.07988 to 2.120	ns	0.0593
	117-Pos vs. 117-Neg	0.9814	0.8882 to 1.075	*	0.0211
	123-Pos vs. 123-Neg	0.8926	0.3350 to 1.450	*	0.0215
	141-Pos vs. 141-Neg	-0.0257	-0.9178 to 0.8664	ns	0.9652
	74-Pos vs. 74-Neg	-0.721	-2.242 to 0.8002	ns	0.1143
**Super block**	122-Pos vs. 122-Neg	2.448	0.1796 to 4.717	*	0.0412
	60-Pos vs. 60-Neg	1.537	-4.029 to 7.102	ns	0.1976
	117-Pos vs. 117-Neg	0.852	0.4825 to 1.221	*	0.0211
	123-Pos vs. 123-Neg	1.092	0.02804 to 2.155	*	0.0469
	141-Pos vs. 141-Neg	-0.08185	-0.7526 to 0.5889	ns	0.4259
	74-Pos vs. 74-Neg	-0.7774	-0.8955 to -0.6592	*	0.0211
**Starting block**	122-Pos vs. 122-Neg	1.74	1.733 to 1.746	*	0.0211
	60-Pos vs. 60-Neg	0.7467	0.1525 to 1.341	*	0.0282
	117-Pos vs. 117-Neg	0.5688	0.02956 to 1.108	*	0.0437
	123-Pos vs. 123-Neg	0.8405	0.2463 to 1.435	*	0.0233
	141-Pos vs. 141-Neg	-0.0414	-1.120 to 1.037	ns	0.9037
	74-Pos vs. 74-Neg	-0.5287	-3.285 to 2.227	ns	0.2808
**Assay buffer**	122-Pos vs. 122-Neg	2.93	-0.4700 to 6.330	ns	0.0679
	60-Pos vs. 60-Neg	2.418	0.5791 to 4.257	*	0.0258
	117-Pos vs. 117-Neg	1.766	0.9842 to 2.549	*	0.0211
	123-Pos vs. 123-Neg	1.937	1.314 to 2.559	*	0.0211
	141-Pos vs. 141-Neg	0.3794	-0.5693 to 1.328	ns	0.1367
	74-Pos vs. 74-Neg	-0.1934	-0.5212 to 0.1345	ns	0.0958
**Pierce buffer**	122-Pos vs. 122-Neg	2.504	0.2653 to 4.742	*	0.0374
	60-Pos vs. 60-Neg	2.094	-0.4095 to 4.597	ns	0.0712
	117-Pos vs. 117-Neg	1.866	-7.581 to 11.31	ns	0.2731
	123-Pos vs. 123-Neg	1.649	0.5807 to 2.718	*	0.0218
	141-Pos vs. 141-Neg	0.1651	-2.699 to 3.029	ns	0.752
	74-Pos vs. 74-Neg	-0.1789	-1.827 to 1.469	ns	0.4714

Supplementary Table 3. Statistical analysis of commercial blocking buffer comparisons with skim milk for IELISAs using positive and negative bovine serum.

	**Tukey's multiple comparisons test (Bovine)**	**Mean Diff.**	**95.00% CI of diff.**	**Summary**	**Adjusted P Value**
**Skim Milk**	122-Pos vs. 122-Neg	2.539	1.866 to 3.211	*	0.0211
	60-Pos vs. 60-Neg	1.876	0.9227 to 2.830	*	0.0211
	117-Pos vs. 117-Neg	0.9572	0.6210 to 1.293	*	0.0211
	123-Pos vs. 123-Neg	1.092	0.6421 to 1.541	*	0.0211
	141-Pos vs. 141-Neg	0.8249	0.5336 to 1.116	*	0.0211
	74-Pos vs. 74-Neg	0.3501	-2.203 to 2.903	ns	0.3837
**Super block**	122-Pos vs. 122-Neg	3.021	-2.670 to 8.711	ns	0.1032
	60-Pos vs. 60-Neg	1.881	1.384 to 2.379	*	0.0211
	117-Pos vs. 117-Neg	0.4903	-0.7879 to 1.769	ns	0.1428
	123-Pos vs. 123-Neg	1.072	0.2378 to 1.905	*	0.0269
	141-Pos vs. 141-Neg	0.4908	-6.588 to 7.569	ns	0.6685
	74-Pos vs. 74-Neg	0.1907	-0.1139 to 0.4952	ns	0.0925
**Starting block**	122-Pos vs. 122-Neg	2.324	-0.3718 to 5.021	ns	0.0679
	60-Pos vs. 60-Neg	1.314	-0.8334 to 3.461	ns	0.0937
	117-Pos vs. 117-Neg	0.4018	-0.3122 to 1.116	ns	0.0988
	123-Pos vs. 123-Neg	1.037	-0.2315 to 2.305	ns	0.0735
	141-Pos vs. 141-Neg	0.4824	-0.5728 to 1.538	ns	0.1186
	74-Pos vs. 74-Neg	0.562	0.3173 to 0.8066	*	0.0211
**Assay buffer**	122-Pos vs. 122-Neg	1.882	1.033 to 2.731	*	0.0211
	60-Pos vs. 60-Neg	1.632	0.6087 to 2.656	*	0.0215
	117-Pos vs. 117-Neg	0.8278	0.2952 to 1.360	*	0.0217
	123-Pos vs. 123-Neg	0.9721	0.1266 to 1.818	*	0.0348
	141-Pos vs. 141-Neg	0.5671	0.3674 to 0.7668	*	0.0211
	74-Pos vs. 74-Neg	0.2585	-4.628 to 5.145	ns	0.7887
**Pierce buffer**	122-Pos vs. 122-Neg	1.846	-0.03490 to 3.726	ns	0.0523
	60-Pos vs. 60-Neg	1.416	-2.071 to 4.903	ns	0.1345
	117-Pos vs. 117-Neg	0.5078	-0.3277 to 1.343	ns	0.0941
	123-Pos vs. 123-Neg	0.6559	-4.234 to 5.546	ns	0.3914
	141-Pos vs. 141-Neg	0.2908	-2.482 to 3.064	ns	0.4855
	74-Pos vs. 74-Neg	0.2285	-3.178 to 3.635	ns	0.6838

Supplementary Table 4. Nucleotide sequence alignment similarity percentages table of capripoxvirus sequences to consensus sequences used for recombinant protein expression.

Accession number	LSDV 60	LSDV 74	LSDV 117	LSDV 122	LSDV 123	LSDV 141
**NC_004002**	98.92	98.97	96.44	97.88	98.64	98.24
MZ577073	99.73	99.07	97.76	99.75	97.87	98.67
MW355944	99.73	99.07	97.76	99.75	97.87	98.67
MW020571	98.92	98.97	96.44	98.39	98.64	98.24
MW020570	98.37	98.97	98.88	98.23	98.26	99.26
MT137384	98.92	98.97	96.44	98.22	98.45	98.24
MT134042	99.73	99.48	97.54	99.41	97.87	99.56
MN072626	98.78	98.97	96.44	98.39	97.67	97.36
MN072625	98.37	98.86	98.21	97.57	97.87	98.97
MN072620	98.37	98.97	98.88	98.23	98.26	99.26
MK441838	99.73	98.86	98.21	99.41	97.87	98.67
KC951854	98.24	98.97	98.88	98.23	98.45	99.12
AF325528	99.73	99.69	97.76	98.9	98.45	99.56

Supplementary Table 5. Amino acid sequence alignment similarity percentages of capripoxvirus sequences to consensus sequences used for recombinant protein expression.

Accession number	LSDV 60	LSDV 74	LSDV 117	LSDV 122	LSDV 123	LSDV 141
**NC_004002**	98.37	98.15	95.3	96.44	99.42	96.02
MZ577073	100	97.83	97.3	97.76	96.49	98.67
MW355944	100	97.83	97.3	97.76	96.49	98.67
MW020571	98.37	98.15	95.3	96.44	99.42	96.02
MW020570	99.18	97.21	97.97	98.88	96.49	98.67
MT137384	98.37	98.15	95.3	96.44	99.42	96.02
MT134042	100	99.07	96.62	97.54	96.49	99.11
MN072626	98.37	98.15	95.3	96.44	96.49	95.13
MN072625	99.18	97.21	97.3	98.21	96.49	97.78
MN072620	99.18	97.21	97.97	98.88	96.49	98.67
MK441838	100	97.52	97.3	98.21	96.49	98.67
KC951854	98.78	97.21	97.97	98.88	96.49	98.22
AF325528	100	99.38	97.3	97.76	97.08	99.11

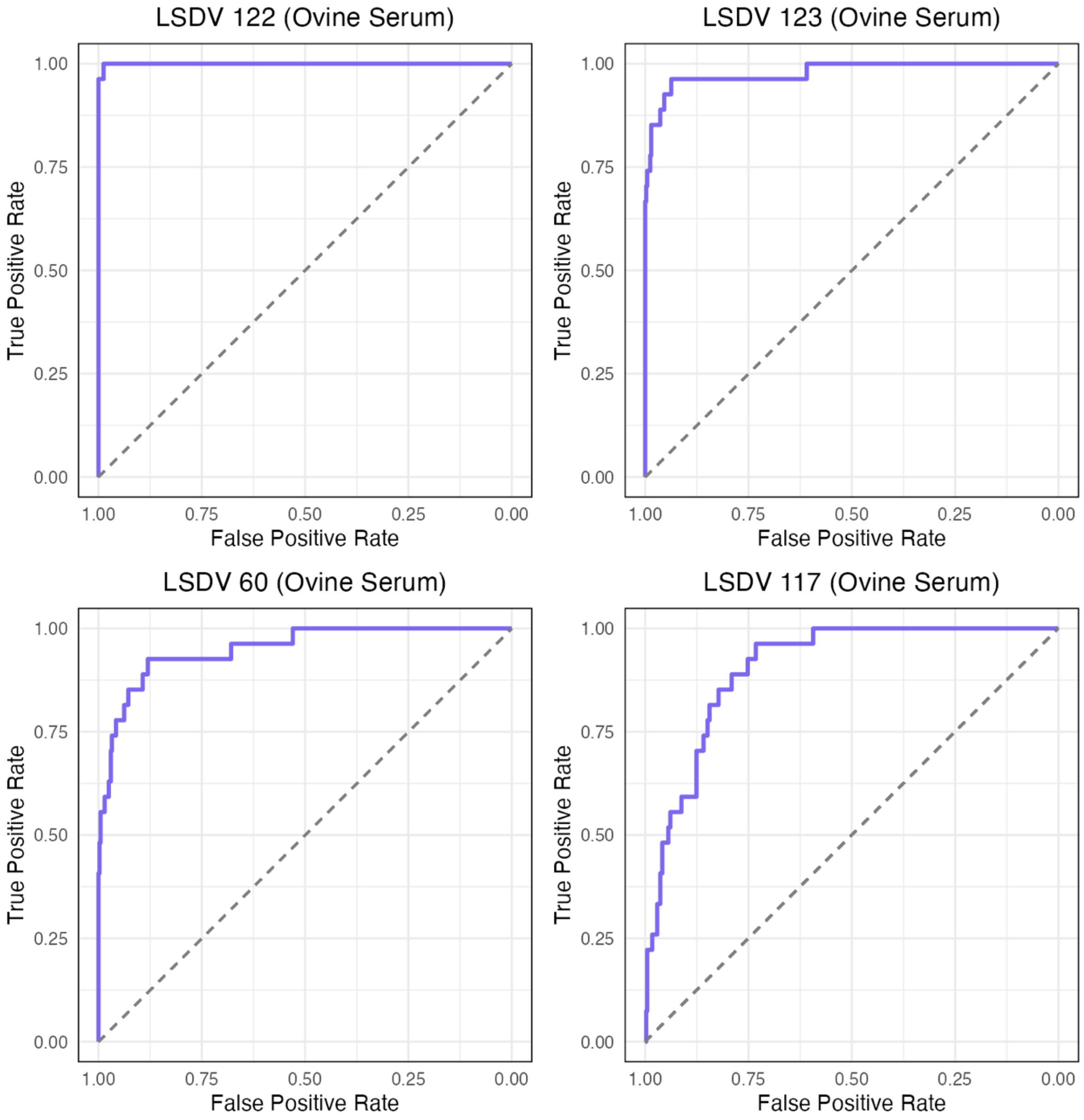

Supplementary Figure 1. Receiver operating characteristic (ROC) curve analysis of ovine indirect ELISAs. LSDV 122 (A33R) has an AUC of 0.9995, P value<0.0001; LSDV 60 (L1R) has an AUC of 0.9503, P value<0.0001; LSDV 117 (A27L) has an AUC of 0.9041, P value<0.0001; LSDV 123 (A34) has an AUC of 0.9783, P value<0.0001.

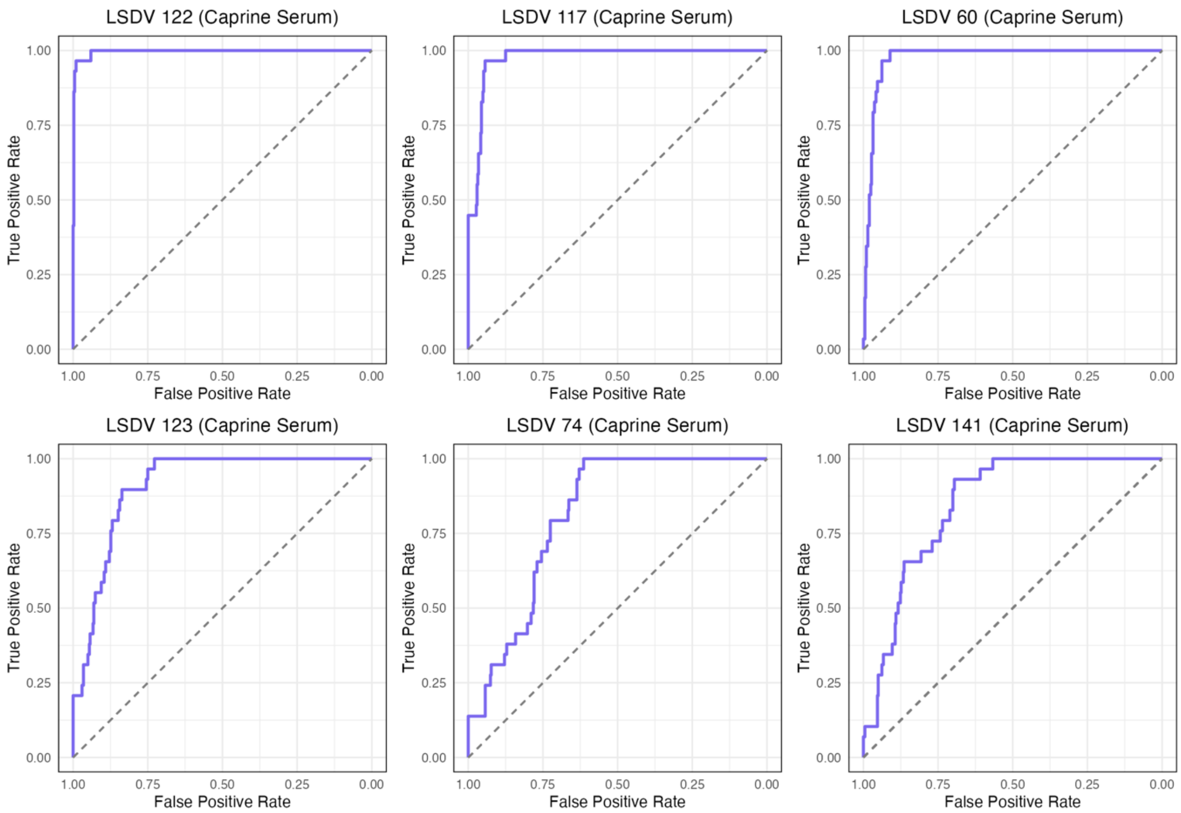

Supplementary Figure 2. Receiver operating characteristic (ROC) curve analysis of caprine indirect ELISAs. LSDV 122 (A33R) has an AUC of 0.9962, P value<0.0001; LSDV 60 (L1R) has an AUC of 0.975, P value<0.0001; LSDV 117 (A27L) has an AUC of 0.9746, P value<0.0001; LSDV 123 (A34) has an AUC of 0.9107, P value<0.0001; LSDV 141 (B5R) has an AUC of 0.8486, P value<0.0001; LSDV 74 (p32) has an AUC of 0.8116, P value<0.0001;

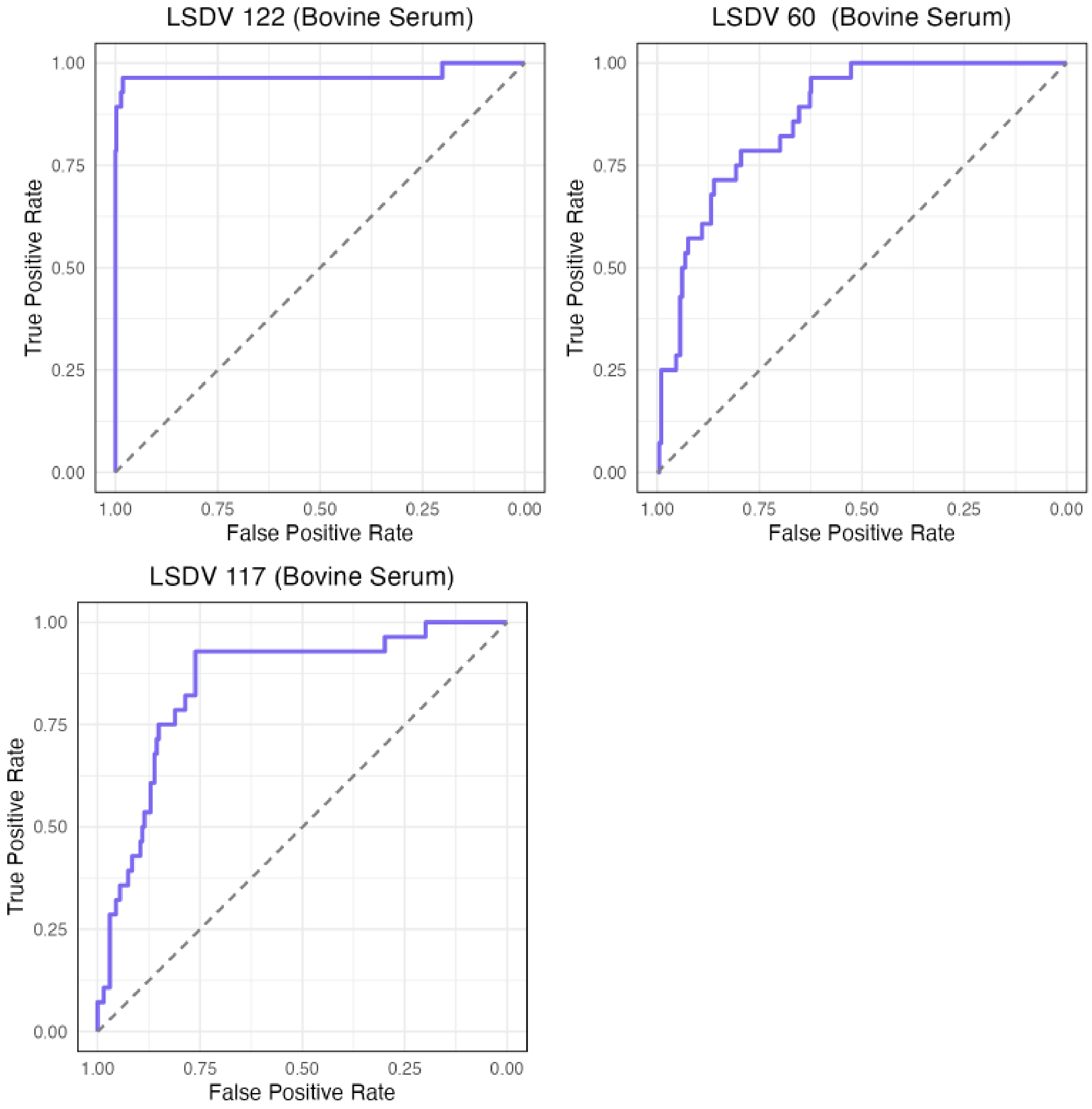

Supplementary Figure 3. Receiver operating characteristic (ROC) curve analysis of bovine indirect ELISAs. LSDV 122 (A33R) has an AUC of 0.9701, P value<0.0001; LSDV 60 (L1R) has an AUC of 0.8677, P value<0.0001; LSDV 117 (A27L) has an AUC of 0.8499, P value<0.0001; LSDV 123 has an AUC of 0.9783, P value<0.0001.

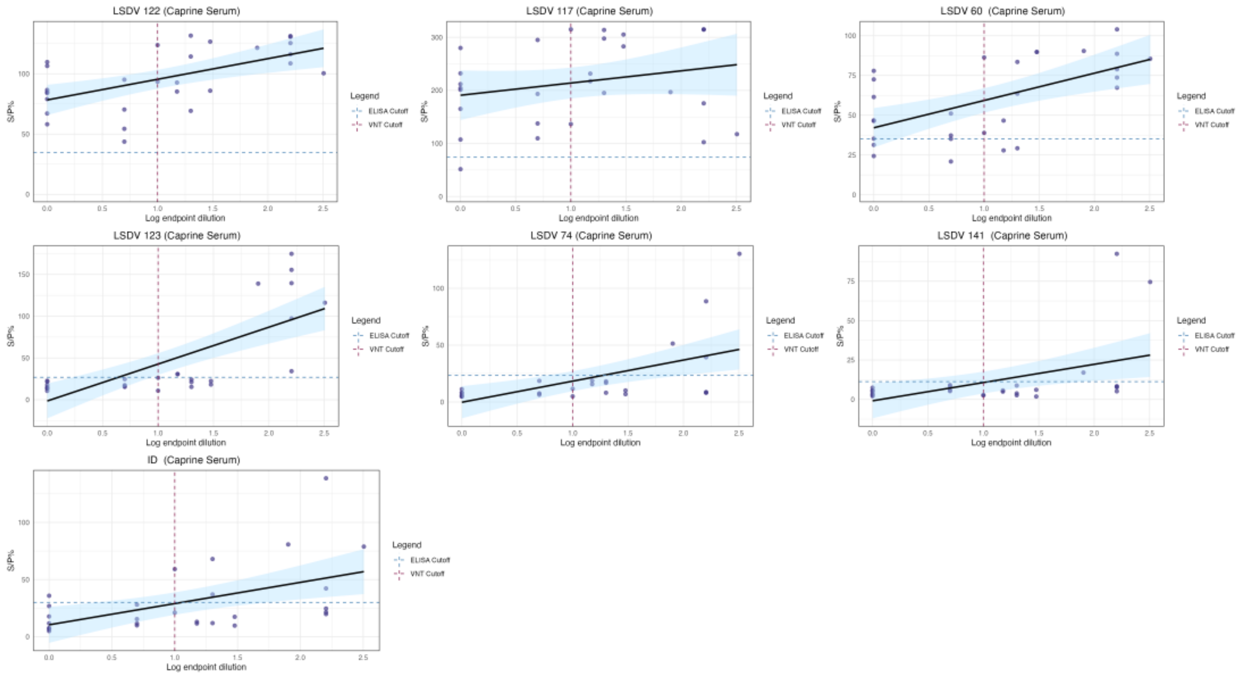

Supplementary Figure 4. Scatterplots of in house developed iELISA and ID ELISA S/P% plotted against VNT results from positive caprine serum. A generalized linear model was used to plot the line of best fit with 95% CI.

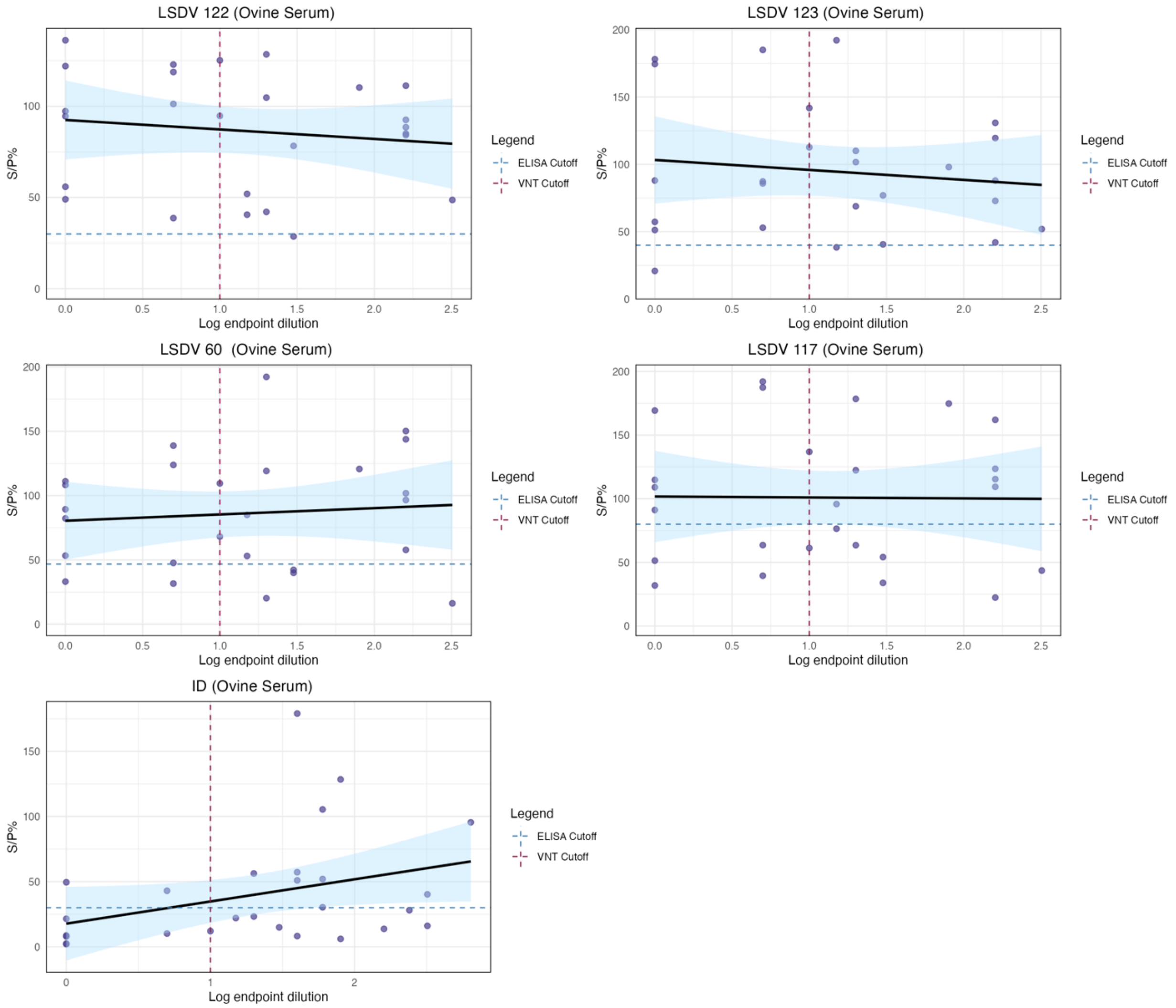

Supplementary Figure 5. Scatterplots of in house developed iELISA and ID ELISA S/P% plotted against VNT results from positive ovine serum. A generalized linear model was used to plot the line of best fit with 95% CI.

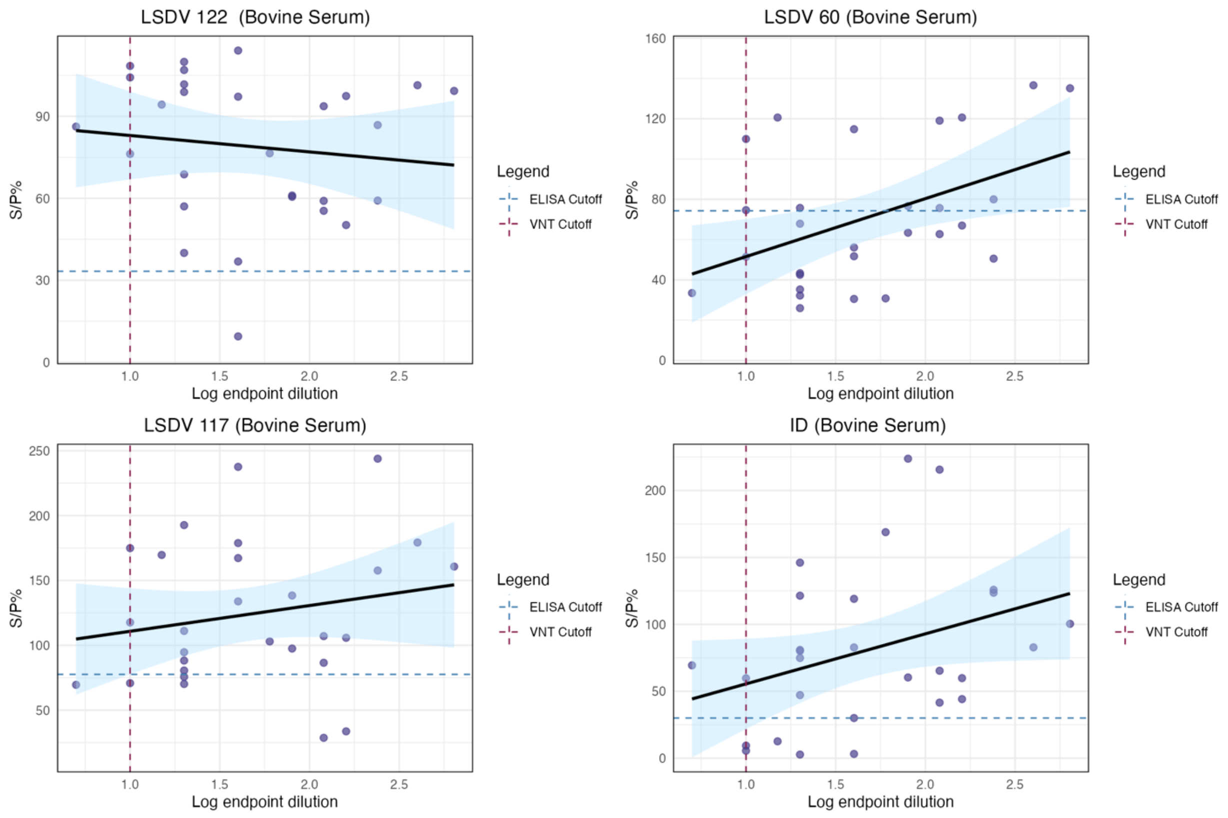

Supplementary Figure 6. Scatterplots of in house developed iELISA and ID ELISA S/P% plotted against VNT results from positive bovine serum. A generalized linear model was used to plot the line of best fit with 95% CI.

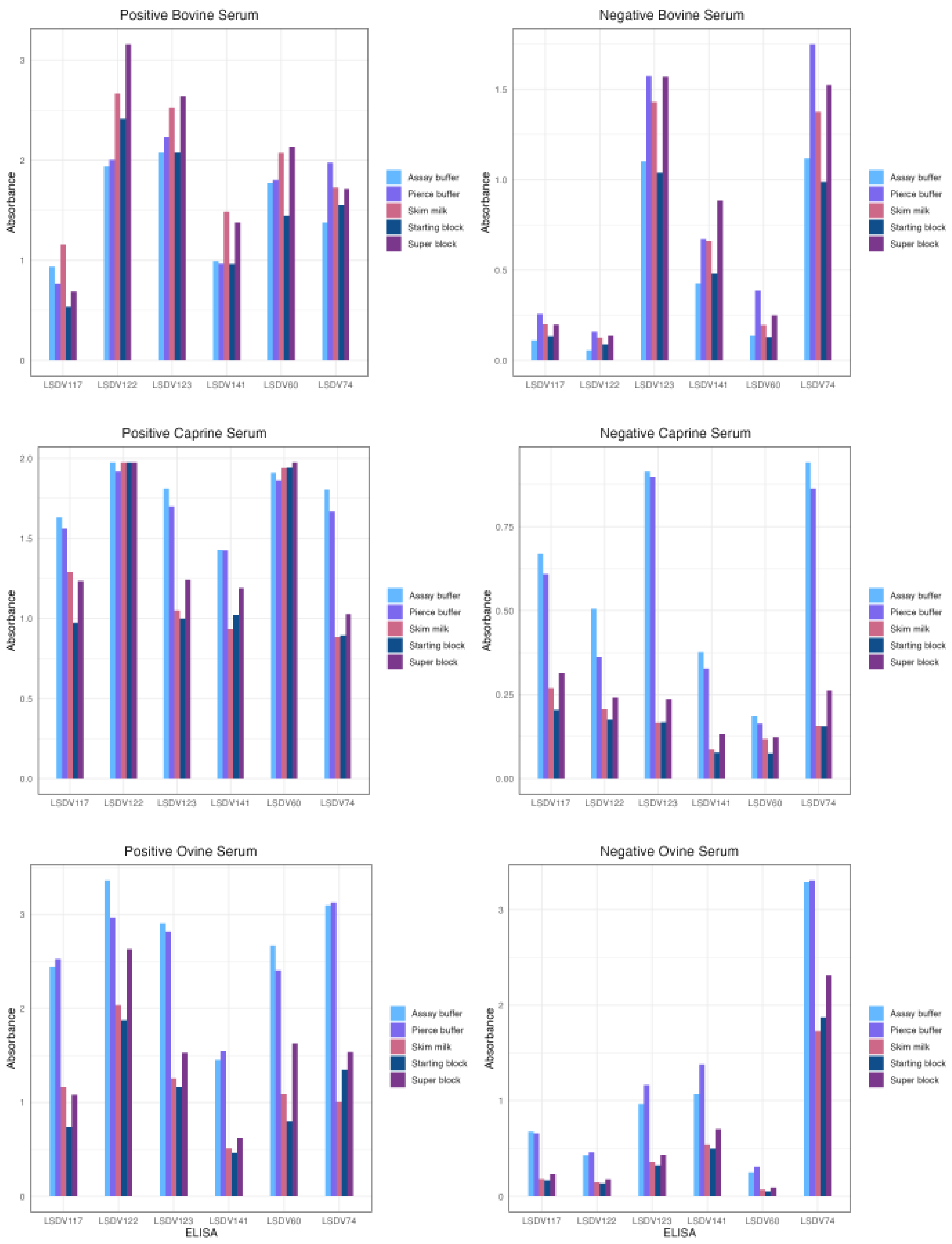

Supplementary Figure 7. Comparison of commercial blocking buffers with skim milk with all recombinant antigens.
